# Quinoid-Thiophene-Based Covalent Organic Polymers for High Iodine Uptake: When Rational Chemical Design Counterbalances the Low Surface Area and Pore Volume

**DOI:** 10.1021/acsami.2c20853

**Published:** 2023-03-16

**Authors:** Onur Yildirim, Arshak Tsaturyan, Alessandro Damin, Stefano Nejrotti, Valentina Crocellà, Angelo Gallo, Michele Remo Chierotti, Matteo Bonomo, Claudia Barolo

**Affiliations:** † Department of Chemistry and NIS Interdepartmental Centre, University of Turin, Via Pietro Giuria 7, 10125 Torino, Italy; ‡ Institute of Physical and Organic Chemistry, Southern Federal University, 344006 Rostov-on-Don, Russia; § Université Jean Monnet Saint-Etienne, CNRS, Institut d’Optique Graduate School, Laboratoire Hubert Curien UMR 5516, F-42023 Saintt-Etienne, France; ∥ INSTM Reference Centre, Università degli Studi di Torino, Via Gioacchino Quarello 15/a, 10125 Torino, Italy; ⊥ ICxT Interdepartmental Centre, Università degli Studi di Torino, Via Lungo Dora Siena 100, 10153 Torino, Italy

**Keywords:** porous organic polymers, covalent organic frameworks, gas storage, iodine, DFT

## Abstract

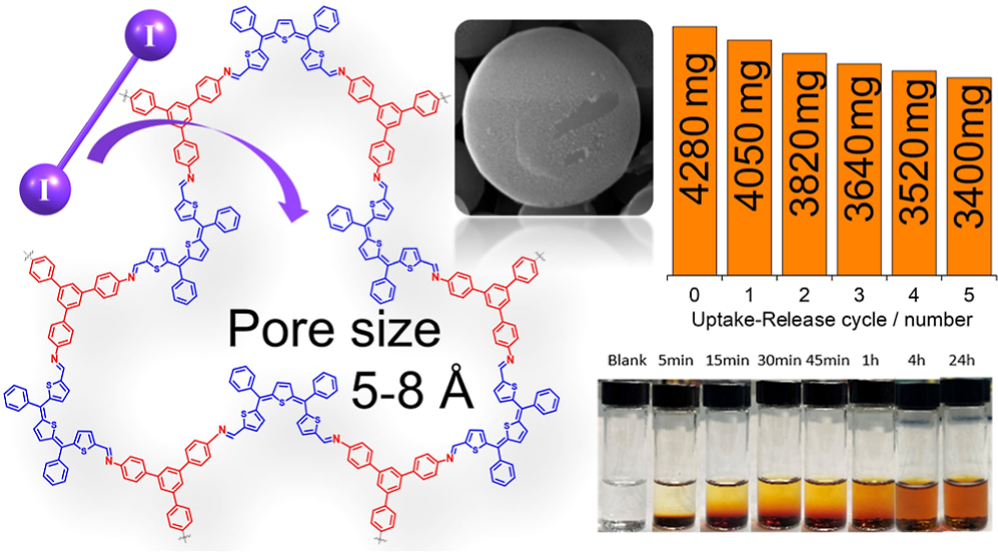

A novel 2D covalent organic polymer (COP), based on conjugated quinoid-oligothiophene (QOT) and tris(aminophenyl) benzene (TAPB) moieties, is designed and synthesized (TAPB-QOT COP). Some DFT calculations are made to clarify the equilibrium between different QOT isomers and how they could affect the COP formation. Once synthetized, the polymer has been thoroughly characterized by spectroscopic (*i.e.*, Raman, UV–vis), SSNMR and surface (*e.g.*, SEM, BET) techniques, showing a modest surface area (113 m^2^ g^–1^) and micropore volume (0.014 cm^3^ g^–1^ with an averaged pore size of 5.6–8 Å). Notwithstanding this, TAPB-QOT COP shows a remarkably high iodine (I_2_) uptake capacity (464 %wt) comparable to or even higher than state-of-the-art porous organic polymers (POPs). These auspicious values are due to the thoughtful design of the polymer with embedded sulfur sites and a conjugated scaffold with the ability to counterbalance the relatively low pore volumes. Indeed, both morphological and Raman data, supported by computational analyses, prove the very high affinity between the S atom in our COP and the I_2_. As a result, TAPB-QOT COP shows the highest volumetric I_2_ uptake (*i.e.*, the amount of I_2_ uptaken per volume unit) up to 331 g cm^–3^ coupled with a remarkably high reversibility (>80% after five cycles).

## 1 Introduction

Nowadays, the demand for energy (∼27 GJ/cap) is increasing to meet human needs.^1^ Among the different possibilities, nuclear power (showing high power density and low carbon emission) has been recently selected by the European Commission in the REPowerEU Plan^2^ as one of the most crucial sources to support “the green transformation of Europe’s energy system” (firmly based on photovoltaic, eolic, hydrogen, and biomasses) to strengthen Europe’s “economic growth, reinforce its industrial leadership, and put Europe on a path toward climate neutrality by 2050”. In this context, uranium is mainly used as nuclear fuel. However, it leads to the production of highly toxic industrial wastes, including radioactive iodine isotopes (^129^I, ^131^I). The latter poses a dramatic health risk when uncrontrollably interacting with the human body, causing thyroid cancer^3^ and fatal diseases in the worst cases.^4,5^ Therefore, its removal from industrial wastes is crucial but still challenging. Some adsorbent materials have been developed to adsorb iodine (such as silica,^6^ chalcogenide aerogels,^7^ activated carbon,^8^ zeolites,^9,10^ and microporous polymers^11,12^) or to transform it into harmless compounds (silver-doped adsorbents^13^). In this context, fully organic materials (and especially covalent organic frameworks (COFs), an emerging class of (crystalline) porous materials) have drawn increasing attention.^14^ For instance, nanoscale channels and regular voids constructed throughout the COF present an ideal environment for storage,^15^ separation, and release processes.^16^ On the other side, the large interface is useful for catalysis^17^ and sensing applications.^18^ Furthermore, the regularity and connectivity of the organic units make COFs promising candidates for applications based on charge carrier transport,^19^ optoelectronics^20,21^ including photovoltaics,^22,23^ electrochemical biosensors,^24^ and electrochemical energy storage.^25−28^ Besides energy-related applications, COFs have been also exploited as substitutes of metallic catalyst in organic reactions^29^ or as adsorbent materials,^30^ especially for hydrogen, methane, or carbon dioxide sequestration.^31^

Iodine, and more broadly gases, uptake in COFs is generally due to their high surface area obtained by controlled crystallinity and remarkably high porosity.^32,33^ A series of 2D COFs were introduced based on the porosity modulation approach for volatile iodine adsorption. COFs exhibited different adsorption capacities according to the pore sizes, and maximum capacity (400 wt %) was reached with a mesopore COF.^34^ As an example, the heteropore 2D COF (SIOC-COF-7)^35^ has two different micropore types with a surface area of 618 m^2^ g^–1^ and a total pore volume of 0.41 cm^3^ g^–1^ and presents a high nitrogen and aromatic rings content, showing an iodine uptake of up to 481 wt %. As a matter of fact, iodine uptake could be improved by introducing heteroatoms within the polymer backbone. Thiophene, thienothiophene, and dithienothiophene or benzotrithiophene are sulfur-rich linkers that have been exploited for this purpose, demonstrating rapid reversible volatile iodine uptake with an adsorption capacity of 276 wt %.^36^ More recently, three different COF adsorbents named TTDP-1, TTDP-2, and TTPD-3 were reported.^37^ In their synthesis, thiophene (TTDP-1), thienothiophene (TTDP-2), and dithienothiophene (TTDP-3) were selected as S-rich linkers and tetraphenyl ethene (ETT) as an additional knot. Adsorption capacities reached were 536, 470, and 425 wt %, respectively.

However, synthetizing highly crystalline and porous COFs is challenging, and the obtained materials suffer from relatively low reproducibility. Aiming at finding innovative functional materials, the synthesis of 2D conjugated microporous polymers (CMPs) is a rising field;^38^ indeed, I_2_ capture ability could be tuned by using thoughtfully designed building blocks embedding specific active sites, extended conjugation, and aromatic units. Furthermore, the synthesis of CMPs with a high surface area would further enhance iodine adsorption.^39^ Unfortunately, only limited I_2_ uptake has been reached with non-crystalline systems. Liao et al. reported on amino-functionalized CMPs with a high adsorption capacity (336 wt %).^40^ Alternatively, CMP was constructed using porphyrin and phthalocyanine as π-electrons-rich building blocks showing an iodine uptake close to 300 wt %.^41^ The I_2_ capture ability could be further increased by coupling aromatic units and positively charged building blocks.^42^ Das et al. synthetized some cationic 2D covalent organic polymers (COP) by using the viologen units crosslinked with hexatopic cyclotriphosphazene core moieties.^43^ Radical cationic (380 and 258 wt %) and cationic (212 and 195 wt %) COPs showed remarkably higher I_2_ uptake compared to the neutral polymers (2.1 and 2.8 wt %), proving the positive interaction between the positively charged moieties and the I_2_ molecules; yet, cationic systems generally suffer from poor stability and very low recyclability.

More interestingly, studies related to porous organic polymers (POPs) indicated that the chemical nature of building blocks is of paramount importance to enhance iodine uptake capacity counterbalancing the lower surface area with respect to crystalline counterparts.^36^ As already discussed for COFs, incorporating electron-rich heterocycles (containing nitrogen and/or sulfur atoms) into the polymer backbone can improve the iodine adsorption capacity due to strong electrostatic interaction between I_2_ and heteroatoms’ lone electron pairs.^12,44^ Recently, Mohan et al. proposed two different POPs based on 1,3,5-triazine-2,4,6-triamine or 1,4-bis-(2,4-diamino-1,3,5-triazine)-benzene and thieno[2,3-*b*]thiophene-2,5-dicarboxaldehyde, obtaining only limited I_2_ absorption (300 wt % at 80 °C) coupled with poor recyclability (<50% after three cycles).^45^

To date, among the reported COFs, 3D-Py-COF shows the highest iodine uptake capacity up to 1670 wt % due to its very high surface area.^46^ On the other hand, among 2D COFs, a tetrathiafulvalene-based one indicated high iodine uptake up to 8.19 g g^–1^ due to the synergistic effect of physical trapping and chemical adsorption of I_2_: its high surface area (up to 2359 m^2^ g^–1^) was designed for efficient physisorption of iodine, and abundant tetrathiafulvalene functional groups were integrated into the COFs allowing efficient chemisorption of the latter. These results show that the presence of (i) aromatic rings in the material backbone, (ii) a high heteroatoms content, (iii) a well-ordered network, as well as (iv) microsphere surfaces could be favorable for high iodine capture. Yet, it should be noted that remarkable I_2_ uptake values (>400 wt %) have been obtained only with materials showing high surface area and/or wide pore dimensions. This partially relegates a thoughtful design of the materials to play a wingman role, especially when high crystallinity could not be reached as for COPs and POPs.

Within this article, we designed and synthesized a linear building block containing quinoid-oligothiophene (QOT) to be exploited as a S-rich linker in an imine-based 2D covalent organic polymer; we used tris(4-aminophenyl)benzene (TAPB) to synthesize TAPB-QOT COP through a Schiff-base reaction. The achievement of TAPB-QOT COP was assessed by means of IR and ^13^C CPMAS solid-state NMR (SSNMR) measurements. All building blocks are designed or selected to contain three different active sites such as π-electrons, sulfur, and aromatic moieties which should assure a promising iodine affinity, counterbalancing the possible low surface area and pore volumes of the synthetized polymers. Density functional theory (DFT) calculations were exploited to both clarify the chemical equilibrium between different isomers of QOT and to support the clarification of the I_2_/TAPB-QOT-COP interactions evidenced by Raman spectroscopy and SEM/EDX morphological analyses. The proposed structure is designed to ensure not only high and fast iodine capture ability (for I_2_ both in its gaseous form and when dissolved in apolar solvents) but also very good recyclability.

## 2 Experimental Section

All experimental details, such as (i) the nature and the purity of the employed materials, the details of (ii) experimental methods and (iii) computational approaches, as well as (iv) the details on the intermediates and final compounds are thoroughly described in Appendix B of the Supporting Information.

## 3 Results and Discussion

### 3.1 COP Synthesis

**Figure 1:**
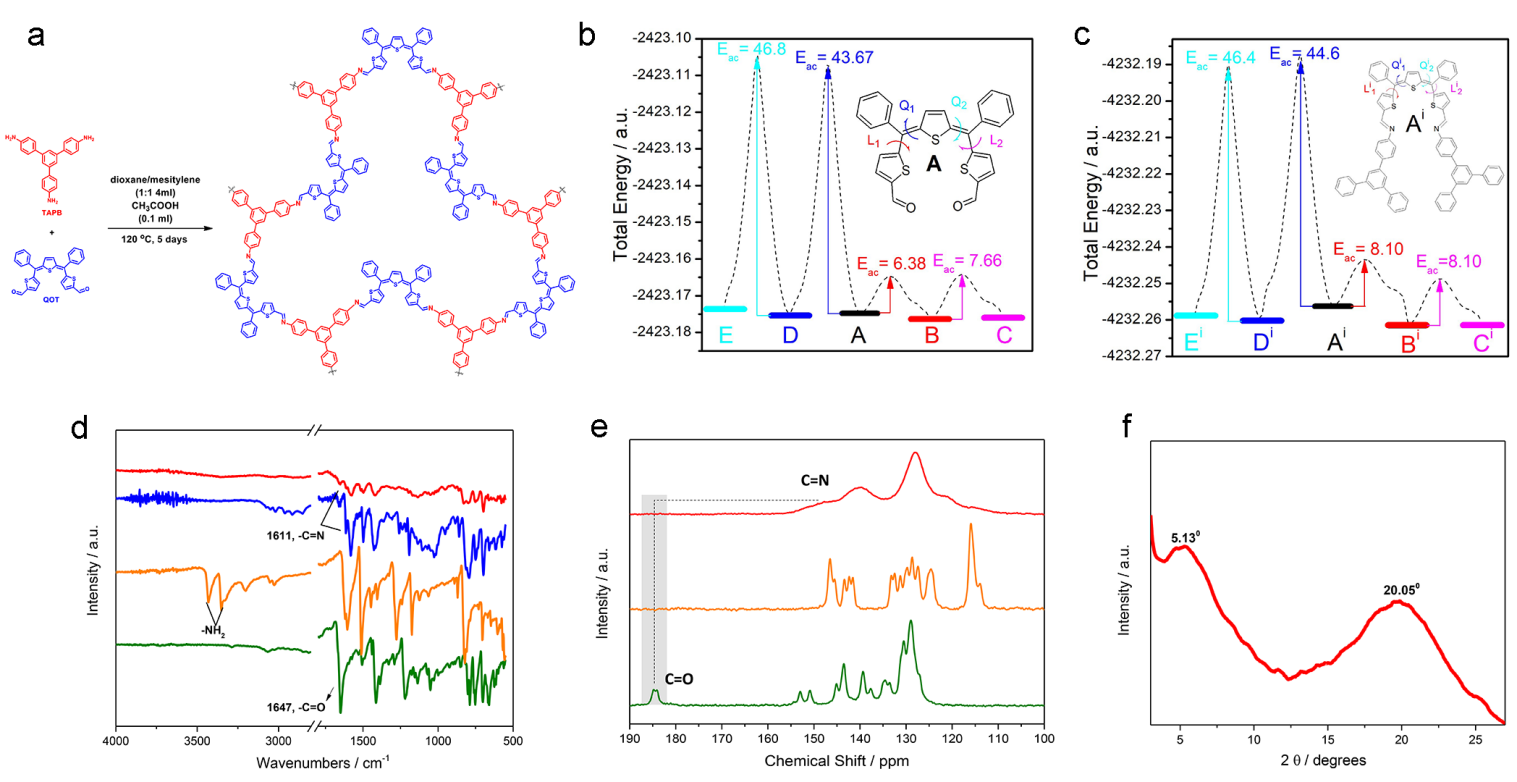
Schematization of the reaction for the synthesis of TAPB-QOT COP (a); energy profiles (dashed line) for forming the isomers of QOT building blocks (b) and COP structural unit (c) by rotation over Q^i^ and L^i^ angles. The activation energy for rotation is calculated in kcal/mol. Infrared spectra of TAPB-QOT COP (red), model compound (blue), TAPB (orange), and QOT (green) in which the spectra are stacked for clarity (d); 1D ^13^C (150.91 MHz) CPMAS experiments, acquired with a spinning speed of 20 kHz, of TAPB-QOT COP (red), TAPB (orange), and QOT (green) in which the spectra are stacked for clarity (e); PXRD pattern of TAPB-QOT COP (f).

A novel 2D covalent organic polymer (TAPB-QOT COP) was synthesized (see Experimental Section for further details and Figure 1a) by a condensation reaction between TAPB and QOT linkers. First, the QOT building block was synthesized (Scheme S1): it has been designed in order to have (i) a high sulfur content, (ii) an extended π conjugated backbone, and (iii) aldehyde moieties to promote the condensation reaction with TAPB; the latter has been selected to obtain a stable polymer and to further extend the π network of the final COP. More interestingly, the designed QOT ligand allows the simultaneous presence of S atoms experiencing two different chemical environments, *i.e.*, in a thiophene geometry and a quinoid one. Albeit (**3**) and its homologues with 4 or 5 thiophene rings have been already proposed as p-type semiconductors,^47^ as far as we are aware, QOT has never been exploited as a building block in covalent organic polymers or frameworks.

The asymmetric building block comprises thiophene and its quinoid form connected by a methine bridge. Due to the asymmetric form of the precursor, isomers’ mixture could form (alkene *Z*–*E* isomers) upon reaction, as proved by ^1^H NMR spectroscopy (Figure S1a). One of the possible isomers seems the most stable one, showing a more intense NMR integral, but two minor peaks (*i.e.*, less likely occurring isomers) could be clearly detected at least. Following some literature reports,^48^ the mixture of isomers was heated (393 K) under reflux using toluene to promote the reorganization of all isomers into the most stable one. Yet, this procedure turned out to be unsuccessful, proving that the energy difference between different forms (at least in toluene solution) is very similar (vide infra). In order to avoid the effect of the solvent (*i.e.*, toluene) that, due to stabilization, could minimize the energy difference between different isomers, the powder was put into a vacuum oven and heated up to 373 K overnight. This approach was successful, as proven by the NMR spectrum (Figure S1b).

To better understand what type of isomers could be formed during the synthesis of the precursor and the energetics behind their interconversion, quantum-chemical calculations were carried out. The optimization of spatial and electronic structures of the six most probable isomers was performed, and the relative energy values were calculated both in the gas phase and in dioxane solution. The results are shown in Figure S2. The isomer B has the minimum total energy both in the gas phase and in solution. The relative energy difference for other isomers is less than 2 kcal/mol. It means that, based on thermodynamics only, all isomers have the possibility to interconvert into one another within the reaction mixture at room temperature, further confirming the NMR results. Therefore, solid conclusions on which isomers could be formed, based on relative energy only, seem unreliable. For this reason, we performed the analysis of kinetic factors for the formation of each isomer. All proposed isomers can be constructed by cis–trans interconversion of two double bonds from the central thiophenes (Q1 and Q2 in Figure 1b) and two single bonds the from terminal thiophenes (L1 and L2 in Figure 1b). The starting structure for the computational analysis was isomer A (inset of Figure 1b) due to its symmetry. By changing the left dihedral angle between central and terminal thiophene rings (L1) from 168° to −21° with a step of 10°, isomer A forms isomer B, and both the energy profile and barrier of rotation could be obtained. The transition-state structure has a dihedral angle of 88°, and the activation barrier of rotation was estimated at 6.38 kcal mol^–1^. The rotation of the right dihedral angle between the central and terminal thiophene rings (L2) of isomer B from 174 to −25° leads to isomer C. The transition state between isomers B and C has a dihedral angle of 88° with an activation barrier at 7.66 kcal mol^–1^. Due to the rotation of structure A over dihedral angle Q1 (from 175 to −7°) isomer D is formed, whereas upon rotation of dihedral angle Q2 (from −178 to −7°) of the latter, isomer E is obtained. Since the link between terminal benzene and central thiophene rings is based on a double bond, the rotation over Q1 and Q2 angles would result in a higher energy consumption. Indeed, the activation barriers for these rotations are 43.67 and 46.80 kcal mol^–1^, respectively, proving that the formation of isomers D and E has sizeable thermodynamic and kinetic limitations. The low relative energy of isomer A and the low enough activation barrier for interconversion from A to B promote the formation of isomer B. The relative energy between isomers B and C is very low (≈0.2 kcal mol^–1^), and the activation barrier is not so big. This favors the establishment of an equilibrium between isomers A, B, and C in solution (relative amount equal to 0.808:0.115:0.077), accounting for the three contributions evidenced in the ^1^H NMR spectrum (Figure S1a). These data prove that, albeit a single isomer is formed after vacuum treatment, the polymerization reaction condition (*T* = 393 K) could induce the formation of other isomers. Therefore, it is worth analyzing the energetic contribution of different isomers toward the COP formation.

For the formation of COP, the suitable conformer of the building block should be obtained but additional energy contribution due to steric hindrance in polymer construction should be considered as well. DFT calculations were carried out (i) to predict the composition of the reaction mixture, (ii) to explore the influence of conformational flexibility on the stability of the low-energy states, and (iii) to study the possibility of interconversions between the six possible isomers (Figure S3). Adding a 1,3,5-triphenylbenzene (TBP) moiety to the QOT leads to an increase in the relative energy in comparison to the sole QOT. The analysis of the relative energy values proves the formation of three isomers B^i^, D^i^, and C^i^. The most favored conformer toward COP formation, following Scheme S1, is isomer C^i^ (Figure 1c). As a matter of fact, isomer B^i^ has the lowest total energy, but isomer C^i^ is only slightly destabilized (≈ 0.2 kcal mol^–1^), which means that these two isomers are in equilibrium (51 vs 49%) at room temperature; indeed, the energy barrier of interconversions between C^i^ from B^i^ is 8.10 kcal mol^–1^. Yet, the COP formation shifts this equilibrium toward isomer C^i^. It is worth noting that isomer D has a quite low relative energy too (less than 1 kcal mol^–1^ more energetic), which means that its existence in the reaction mixture is possible. However, the content of isomer D^i^ could be lower due to higher relative energy and activation barrier of rotation. The formation of isomer E^i^ is less favorable since there are thermodynamic (highest relative energy) and kinetic (high activation barrier for rotation) limitation factors.

Based on these analyses, isomers B^i^ and C^i^ are the most likely to be present in the reaction mixture, whereas only a very small fraction of D^i^ (limited by the extremely high energy barrier in passing from A^i^ to D^i^) can exist, thus preventing the formation of an ideal COP crystal according to the reaction pathway. Isomer B^i^ is not suitable to construct a 2D covalent organic polymer because it has asymmetric and highly hindered structure. Two other possible forms (E^i^, F^i^) cannot be suitable because they cause distortion in the bulky COP structure. On the other side, the most likely form is C^i^ because its relative energy is much lower than that of others and the geometry is more appropriate to build a 2D polymer.

Pore size and homogeneity of their distribution are two of the most important properties of COPs, especially considering their possible application as I_2_-sponges. Aiming at an estimation of the pore size, we optimized the structural unit of studied COP C^i^ (Figure S4) from which it appears clear that the optimized geometry is not completely flat; on the other hand, it should be recalled that when multilayered systems are conceived, a lot of weak interlayer interactions could take place forcing the flattening of the structural unit.^49,50^ For a more specific estimation of the pore area, the latter has been divided into 10 triangles (Figure S5a). Polygon vertexes are sulfur atoms of the thiophene ring and the carbon atom of the benzene ring, and dummy atoms are in the center of the C–C bond of the thiophene ring. The area of the triangle was calculated according to the following equations
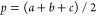
1
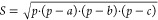
2where *a*, *b*, and *c* are the lengths of the sides of the triangles, *p* is the semiperimeter, and *S* is the area of the triangle. The pores area of the structural unit based on the sum of the area of a triangle is 11.7 nm^2^. Since the deviation from the flat structure of the optimized unit is sizeable (Figure S4), a displacement of roughly ±1 nm^2^ could be expected in the real polymer when a crystalline structure is considered. Pores size distribution calculated using the DFT approach reveals mainly the presence of micropores with an average pore diameter of around 1.3–1.5 nm (Figure S5b). The computational sources for quantum-chemical calculations based on DFT are very sensitive to the size of the studied system. In the presented results, the structural unit consists of 546 atoms, which is a relatively high number of atoms in running the calculation. Therefore, albeit the study of interlayer interaction or several numbers of structural units by DFT would be very informative, it became a very challenging task. For this calculation, it would be more reasonable to use theories based on molecular dynamic approaches which will be the subject of our future work.

### 3.2 COP Characterization

Once the thermodynamics and the kinetics behind the formation of the isomer mixture were investigated (obtaining the formation of one single isomer after vacuum treatment), we proceeded with the synthesis of COP. Due to the relatively low solubility of COPs in conventional solvents, the occurrence of the reaction was first monitored by infrared spectroscopy. Following on from the polymer formation, a complete disappearance of N–H stretching bands at 3424, 3347, and 3187 cm^–1^ was expected (Figure 1d); indeed, the −NH_2_ moieties of TAPB reacted with the carbonyl groups of the QOT, leading to the formation of imine bonds in the polymer as proved by the rising of a band due to the C=N stretching (1611 cm^–1^). Indeed, the carbonyl stretching band (1647 cm^–1^) is absent in the spectrum of the polymer. To verify the completion of the reaction and the formation of TAPB-QOT-COP, we also performed SSNMR spectroscopy. Indeed, this technique is very sensitive to carbonyl-to-imine transformation. A chemical shift disappearance of the carbonyl signal (at 185.5 ppm) of the QOT in TAPB-QOT COP spectrum suggests a complete conversion of the carbonyl group into an imine group, clearly confirming the formation of the COP (Figure 1e). Indeed, according to the formation of an imine C=N group, the signal shifts to lower chemical shifts, around 150 ppm, overlapping with the aromatic resonances. It should be noted that the signals of COP are considerably wide, and this suggests a high degree of amorphousness (as proven by PXRD data, *vide infra*). At the same time, the high degree of disorder of the sample makes the acquisition of a ^15^N spectrum of TAPB-QOT COP prohibitive. Indeed, ^15^N is present only in 0.36% at natural abundance, resulting in a major sensitivity penalty. Sensitivity is made even worse by its low gyromagnetic ratio (γ = −27.126 × 10^6^ T^–1^ s^–1^), which is 10.14% of that of ^1^H. Thus, the only ^15^N CPMAS spectrum acquired was that of TAPB and is reported in the Supporting Information (Figure S6).

After checking the nature of the obtained material via both IR and NMR spectroscopy, powder X-ray diffraction (PXRD) was employed to determine the structural regularity of TAPB-QOT-COP. The PXRD pattern demonstrates two broad diffraction peaks at around 5.1° and 20.5° that might be corresponding to the (100) or (110) and (001) planes, respectively (Figure 1f).^51^ Yet, peaks are relatively broad showing the occurrence of low-range crystallinity due to the random displacement of the 2D layers (*i.e.*, exfoliation).^52^ These findings agree with the computationally predicted relatively low energy differences for isomers leading to a mixture of COP with slightly different geometries, that would severely reduce the crystallinity of the structure making less favorable the packing of isostructural layers.

The thermal stability of the TAPB-QOT polymer was investigated by thermogravimetric analysis (TGA), as shown in (Figure S7). A moderate weight decrease was recorded after 523 K likely due to the evaporation of solvent molecules trapped into pores. Indeed, the synthetized COP seemed to be highly stable up to 673 K (93% residual), and it still retained around 60% weight at 1073 K. Exhibiting such high stability is significant in terms of using it as adsorbent material even in harsher conditions compared to room temperature.

As widely demonstrated in the literature, high surface area polymers are very effective in I_2_ uptake due to the great availability of active sites and the possibility of physically trapping I_2_ into the pores. For this reason, a careful evaluation of the textural properties of the TAPB-QOT-COP polymer was carried out by collecting adsorption/desorption isotherms with different molecular probes. A first attempt was made employing N_2_ at 78 K. The adsorption/desorption isotherm collected in this condition is reported in Figure S8. The material exhibits a very low N_2_ adsorption capacity, basically approaching zero, and the computed BET specific surface area (SSA) is less than 10 m^2^ g^–1^.

**Figure 2:**
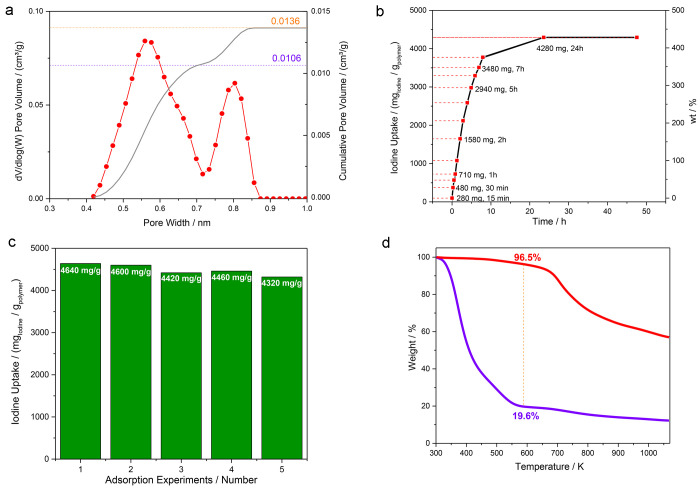
Cumulative pore volume (grey) and pore size distribution (red) of TAPB-QOT-COP calculated by NL-DFT from CO_2_ adsorption isotherm at 273 K (a); gravimetric measurement of iodine uptake (mass polymer = 10 mg) at 350 K and 1 atm (b); repetition of iodine uptake (same conditions as Figure 2b) for pristine samples (c); comparison of thermogravimetric curves of I@TAPB-QOT COP (violet solid line) and pristine TAPB-QOT COP (red solid line) (d).

It is well known that nitrogen adsorption is of limited value for the characterization of microporous materials due to kinetic restrictions at a cryogenic temperature (77 K). Indeed, the restricted diffusion prevents nitrogen molecules from entering the narrowest micropores.^53,54^ The diffusion limitations of nitrogen in ultra-micropores can be overcame by the use of CO_2_ at 273 K, thanks to the enhanced diffusion kinetics of this small probe molecule at a temperature closer to ambient. The CO_2_ adsorption/desorption isotherms measured at 273 K on TAPB-QOT are shown in Figure S9. The amount of adsorbed CO_2_ is higher compared to N_2_ at 77 K, and the BET SSA computed by the CO_2_ adsorption isotherm is 113 m^2^ g^–1^. Moreover, by applying the non-local DFT method, as reported in the Experimental Section, the cumulative pore volume curve was derived and is reported in Figure 2a (grey curve), allowing the calculation of the TAPB-QOT-COP micropore volume (0.014 cm^3^ g^–1^). The pore size distribution (PSD) was then obtained and is reported in Figure 2a (red curve). The PSD has a bimodal character, with two maxima located at 0.6 and 0.8 nm, with a clear prevalence of the smaller micropore family (0.010 cm^3^ g^–1^), as derived by the cumulative pore volume curve. The two types of micropores obtained by the non-local DFT (NL-DFT) are in fair agreement with the expected TAPB-QOT-COP porous structure and relatively lower compared to highly ordered systems.^55^ One should note that the micropore size obtained from CO_2_ adsorption data is slightly lower compared to the values obtained by theoretical calculations (Figure S5). This behavior could be ascribable to the poorly crystalline nature of the polymers partially preventing the gas access to inner microcavities: as a matter of fact, the unsymmetrical stacking of the different 2D COP layers leads to a displacement of the cavity in the *z*-axis causing a reduction of the effective pore dimension. A slight discrepancy between the theoretical and experimental value could be related to the selection of the proper model in the NL-DFT elaboration of experimental data. Indeed, despite the high number of COF structures reported to date, proper DFT kernels to study the unique pore size and shapes of these microporous materials are still missing. For this reason, the experimentally obtained PSD could be affected by an error due the selection of the proper kernel of isotherms. However, although the NL-DFT analysis was carried out considering a slit pore geometry and applying a model for CO_2_ adsorption at 273 K on carbons, the fit between the calculated and the experimental isotherm was perfect, with a very low standard deviation (around 0.00925 cm^3^ g^–1^ STP) as reported in Figure S10, testifying the goodness of the experimental PSD.

Despite the relatively low surface area and pore volume values, the thoughtful design of building blocks having (i) a bent form and (ii) S-, N-, and aromatic sites (active toward I_2_ interaction) makes the exploitation of the polymer as an iodine sponge promising. To evaluate the adsorption capacity of TAPB-QOT COP, a gravimetric approach (see Supporting Information for more details) was employed. The iodine-uptake kinetic of TAPB-QOT was proven to be remarkably fast: the amount of adsorbed I_2_ constantly rose (mean slope of 500 mg/h) for the first 7 h before reaching a plateau, having maximum absorption capacity of 428 wt % after 24 h (Figure 2b) that reaches 464 wt % after 48 h. A very high reproducibility of the iodine uptake was obtained (449 ± 11 wt %,Figure 2c). As far as we know, the iodine capacity of TAPB-QOT COP is comparable or even higher than the highest capture values reported in the literature for POPs (see Table 1).^32^ Furthermore, the dramatic effect of the presence of heteroatoms in the backbone of the polymers is evident when the iodine uptake (*i.e.*, 4640 mg g^–1^) is normalized for the pore volume of the COP (*i.e.*, 0.014 cm^3^ g^–1^) to obtain the volumetric uptake of I_2_ (*i.e.*, 331 g cm^–3^) that is the amount of iodine captured for a unit of volume. The latter is the highest value ever reported for POPs (see Table 1). This finding is even more disrupting, considering that the overall pore volume is generally quite underestimated if N_2_ (literature reports) is employed instead of CO_2_ (this paper).

**Table 1:** Iodine Adsorption Capacities in Selected POP Materials

materials	BET surface area (m^2^ g^1^)	pore size (Å)	pore volume (cm^3^ g^–1^)	iodine uptake (wt %)	volumetric iodine uptake (g cm^–3^)	references
TPB-DMTP	1927	33	1.28	620^a^	4.84	(56)
TTDP-1	12.08	17.9	0.036	536^a^	149	(37)
CMPN	86.2	101	0.218	502^a^	23.0	(57)
TTA-TTB	1733	22	1.01	500^a^	4.95	(56)
SIOC-COF-7	618	11.8	0.41	481^a^	11.7	(35)
CalPOF-1	303			477^a^		(58)
ETTA-TPA	1822	14, 27	0.95	470^a^	4.95	(56)
**TAPB-QOTCOP**	**10**			**464**		**this work**
**TAPB-QOTCOP**^b^	**113**	**5.6–8**	**0.014**	**464**^a^	**331**	**this work**
NDB-H	116.93	74.6	0.13	443^a^	34.1	(59)
NDB-S	56.45	75.5	0.11	425^a^	38.6	(59)
TTDP-3	13.20		0.052	470^a^	90.4	(37)
CalPOF-2	154			406^a^		(58)
POP-2	41	20, 400		382^a^		(60)
COP_1_^0^				380^c^		(43)
POP-1	12	20, 400		357^a^		(60)
CalPOF-3	91			353^a^		(58)
SCMP-II	119.76	20		345^a^		(61)
HCMP-3	82	<10	0.08	336^c^	42.0	(40)
HCMP-1	430	<10	0.22	291^c^	13.2	(40)
AzoPPN	400	5.8–12.7	0.68	290^a^	4.26	(41)
CMPNH_2_	6.44	63	0.012	283^a^	236	(57)
HCMP-2	153	<10	0.06	281^c^	46.8	(40)
COP_2_^0^				277^d^		(43)
PAF-24				276^a^		(42)
BTT-TAPT-COF	864	10–20	0.56	276^a^	4.92	(36)
PAF-23				271^a^		(42)
TTA-TFB	1163	16	0.55	270^a^	4.91	(56)
PAF-25				260^a^		(42)
NAPOP-4	626	12.7	0.15, 1.17	265^a^	17.6	(62)
COP_2_^++^				258^d^		(43)
NAPOP-3	702	<4.3, 5.8	0.18, 1.01	241^a^	13.4	(62)
NAPOP-2	458	<4.2, 5.9	0.10, 0.78	239^a^	23.9	(62)
SCMP-2	855		1.50	222^a^	1.48	(12)
HCMP-1		<10		222^c^		(40)
COP_1_^++^				212^d^		(43)
COP_1_^.+^				211^d^		(43)
CMP-4	9.5	4.6–8.2		208^a^		(63)
NAPOP-1	657	<3.9, 7.2	0.25, 1.49	206^a^	8.24	(62)
CMPH	222.4	38	0.213	195^a^	9.15	(57)
CMP-2	20.2	4.6–8.2		177^a^		(63)
CMP-1	16.1	4.6–8.2		151^a^		(63)
CMP-3	284.6	4.6–8.2		131^a^		(63)

a Iodine vapor adsorption, 75–80 °C, ambient pressure.

b Measured by CO_2_ absorption at 273 K.

c Iodine vapor adsorption, 358.15 K, 1.0 bar.

d Iodine vapor adsorption, 60 °C, ambient pressure.

Some thermogravimetric analyses were performed to further quantify the iodine uptake; indeed, the weight loss occurring below 573 K in the I@TAPB-QOT polymer could be ascribable to the thermal-induced I_2_ release, and this data could be compared to the ones obtained through the gravimetric approach. The weight loss of I@TAPB-QOT-COP between 323 and 573 K (measured as the difference between the weight of I@TAPB-QOT-COP polymer and the pristine form at 573 K) is around 80% (Figure 2d), leading to a calculated I_2_ uptake value as high as 4000 mg g^–1^ (400 wt %), comparable with iodine adsorption results (Figure 2b,c).

**Figure 3:**
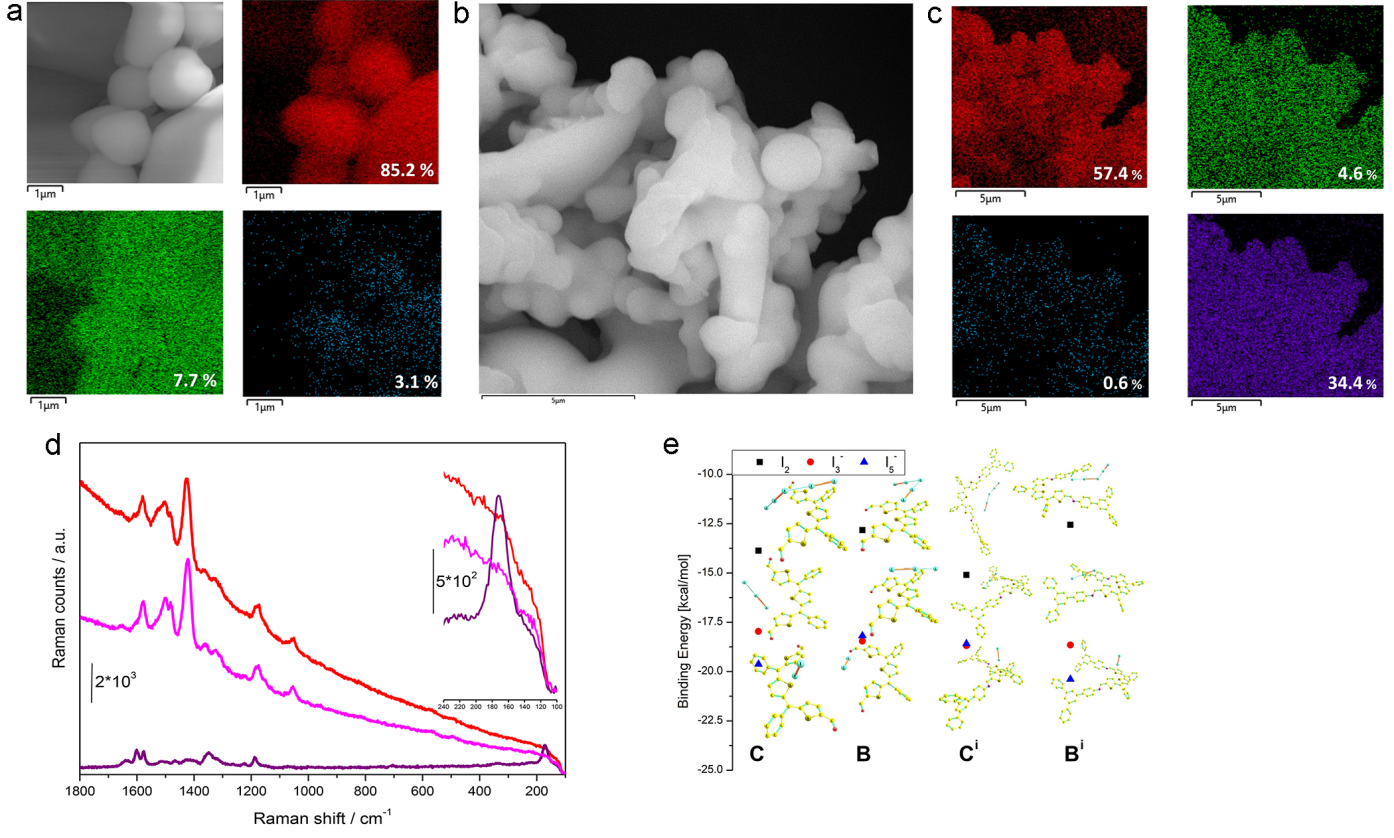
SEM images of TAPB-QOT-COP and elemental mapping measured by EDX in which C, S, and N are in red, green, and blue, respectively, whereas the relative weight ratio is reported in white (a); SEM image of I@TAPB-QOT COP (b); elemental mapping measured by EDX of the sample reported in Figure 3b, in which C, S, N, and I are in red, green, blue, violet, respectively, whereas the relative weight ratio is reported in white (c); Raman spectra of TAPB-QOT-COP (red), I@TAPB-QOT COP (violet), and TAPB-QOT COP-Rel (magenta), in the inset is reported the zoomed portion between 100 and 240 cm^–1^ (d); density functional theory calculations of the binding energies of C, B, C^i^, and B^i^ isomers of COP building block with I_2_, I_3_^–^, and I_5_^–^ (black squares, red circles, and blue triangles, respectively). The structures were obtained by geometry optimization without any symmetry constraints (e).

To more precisely investigate the role of the heteroatoms in I_2_ uptake, we collected some SEM images of I@TAPB-QOT COP (also correlated by EDX analyses), comparing them with the pristine polymer. TAPB-QOT-COP showed a morphology composed of uniformly agglomerated spheres with different sizes, and the EDX pattern evidences the presence of C, S, and N as constitutive elements (Figure 3a). Following on from the I_2_ uptake, polymer nanospheres tend to agglomerate (Figure 3b) due to the loading of iodine onto the polymer surface; additionally, a typical signal of iodine could be seen in the EXD mapping (Figure 3c). Very interestingly, an almost perfect superimposition of the I and S mapping could be detected; more importantly, the ratio between the atomic percentage of S and I is approximately two, proving that sulfur atoms are the ones that likely bind I_2_ molecules. In order to further verify this, we also performed Raman measurements adopting the 514 nm exciting laser line (*i.e.*, in resonant conditions with I_2_ molecule, *vide infra*); the results are shown in Figure 3d, where Raman spectra recorded on pristine COP (TAPB-QOT-COP, solid red line), I_2_-contacted COP (I@TAPB-QOT-COP, solid purple line), and after two I_2_ release cycles (TAPB-QOT-COP-Rel, solid magenta line) are presented. When Raman spectra obtained on TAPB-QOT-COP and I@TAPB-QOT-COP are compared, three main features can be noticed: (i) the appearance of a strong band (hereafter ν_I_2__) located at 172 cm^–1^ (see also inset in Figure 3d); (ii) a strong modification in the 1700–1100 cm^–1^ range after I_2_ addition; and (iii) a quenching of the fluorescence background characterizing the TAPB-QOT-COP Raman spectrum. As far as feature (i) is concerned, ν_I_2__ is located quite near (even if remarkably red-shifted) in a Raman shift range compatible with the I_2_ stretching vibrational mode which in solid/gas phase is centered at 180/214 cm^–1^ with A_g_/A_1g_ symmetry;^64^ this testifies that I_2_ uptake by COP can be followed by Raman spectroscopy too. It is worth noticing here that a band in the same position appears after a 12 mM solution of I_2_/*n*-hexane is added of 3-hexylthiophene (Figure S11): this is accompanied by the consumption of the strong and sharp peak centered at 214 cm^–1^ (*i.e.*, quite close to what observed for isolated I_2_ molecules in the vapor phase), suggesting the formation of a I_2_-3-hexylthiophene adduct, thus enforcing the observation coming from EDX analysis that a one to one I_2_/S interaction is occurring.

In particular, the interaction between I_2_ and TAPB-QOT-COP is expected at a higher energy than what is observed for I_2_ entrapped in TiO_2_ nanovoids, where ν_I_2__ falls at 178 cm^–1^.^65^ Also, the selective formation of I_2_/QOT adducts is strongly supported by the absence of signals in the 214–180 cm^–1^ range (see solid purple line in Figure 3d), which should be indicative for the formation of non-interacting (I_2_)_*n*_ (2 < *n* < 7) small clusters.^64^ The release of iodine leads to an almost complete recovery of the spectrum of the pristine material but with a lower background ascribable to a decrease of the fluorescence of the spectrum, probably due to the presence of some iodine molecules still entrapped in the COP backbone, as confirmed by the uptake/release study (*vide infra*). Such evidence is also corroborated by computational data (Figure S12) in which the rising of a band at around 150–160 cm^–1^ and the lowering of the intensity of the band at 1425 cm^–1^ could be clearly seen following on from the binding of an iodine molecule.

To estimate the binding energy of TAPB-QOT-COP structural units with iodine systems, the optimization of electronic and spatial structures was done. As iodine sources, three types of systems were used (molecular form I_2_ and two anionic forms I_3_^–^ and I_5_^–^). It was found that the main absorption site is the central thiophene ring (*i.e.*, the thiophene in its quinoid form) and the molecular form of iodine localizes perpendicular to the heterocyclic ring. Similar binding geometry was published earlier.^66−68^ The localization of anionic forms has two types. The first one consists of their stabilization above or under COP-structural units (isomers A, B, D, F). In the second localization geometry, the iodide ion lies in the same plane as the COP structural unit, inside a pocket formed by three thiophene rings (C and E isomers). This second type of binding is more suitable for iodine uptake in COP since adsorbed systems will be localized in the polymer pores and will not interfere with the formation of a layered structure of COP. In the case of the small COP structural units, there are no significant iodine binding energy differences. The values are in the ranges of 10–14, 16–21, and 14–20 kcal mol^–1^ for I_2_, I_3_^–^, and I_5_^–^, respectively. The results for the two most interesting isomers (*i.e.*, C and E) are shown in Figure 3e, whereas the others in Figures S13–S18. The isomer C has one of the biggest binding energies with I_2_ and I_*n*_^–^, suggesting a markedly stronger affinity of this isomer toward iodine species. The obtained results are consistent with expectations and explain the experimental observation that, among all COP structural isomers, isomer C exhibited more suitability for COP formation and a higher adsorption capacity of iodine sources.

Albeit the above-discussed evidence clearly points toward a pivotal role of thiophene (and mainly its S atom in the quinoid form) in iodine uptake, an effective role of benzene rings (as electron-rich moieties) could not be completely ruled out. Once more, Raman spectroscopy proved to be a very useful tool to investigate the I_2_-COP interaction: this technique has already been adopted in the past to study the interaction of iodide with electron donor systems.^69^ Indeed, I_2_-benzene complexes (as emerging from the literature data^70,71^) are characterized by a small red shift (near 2 up to 6 cm^–1^) of the frequency associated to I-I stretching which in the gas phase falls at 213 cm^–1^. The same holds for I_2_–C=C complexes (*e.g.*, I_2_ stretching frequency at 199 cm^–1^ for I_2_–1-hexene complex^70^). Conversely, the red shift of I_2_ stretching frequency observed on the present COF system is quite bigger (I_2_ stretching frequency moves from 213 to nearby 170 cm^–1^) and comparable with what is observed for 3-hexyl-thiophene (Figure S11). Such an interpretation is further corroborated by the comparison of Raman spectra of I@TAPB-QOT COP, pristine COP, and its precursors: in fact, QOT vibrational features appears to be strongly quenched after I_2_ adsorption, whereas TAPB features remain quite unaffected (Figure S19). Therefore, the role of the benzene rings could not be completely ruled out, but the latter seems to be marginal compared to the one assured by the S-rich moieties.

Further support in this interpretation comes from calculations we made to better evidence the charge delocalization in the pristine and I_2_-loaded COPs. To reduce the computational time, we simulated the model compounds (A′–F′), but such approximation would not impact result reliability. To minimize the possible selection of a local minimum energy point, the iodine was located at the center of the model compounds. Then, we extracted Mulliken atomic charges of all electron-rich moieties (*i.e.*, S, N, and benzene rings) from all different isomers before and after the I_2_ absorption. As one can see from Table S1, the interaction leads to charge redistribution mainly on the S atoms, whereas only negligible changes could be seen for N (Table S1) or the C of the benzene rings (Tables S2 and S3), further proving their marginal (*i.e.*, supporting) role in the uptake process. It should be noted that useful, complementary information could be extracted by ^127^I NMR or ^33^S NMR, the chemical shift being strongly influenced by the charge state. Unfortunately, liquid-phase NMR was impossible to perform due to the very low solubility of TAPB-QOT-COP; on the other hand, SS-NMR of both the nuclei is very challenging: ^127^I SSNMR spectra are hard to interpret due to the breakdown of second-order perturbation theory.^72^ Indeed, even for a single site in a crystalline sample, the linewidths broaden in the range of MHz and this will be worsened by the partial amorphousness of our samples and by the presence of slightly different I_2_-coordination geometries. Concerning the ^33^S SSNMR, the receptivity relative to ^13^C at natural abundance is 0.101 which is even further complicated by its quadrupolar nature. Having this in mind, we performed ^13^C CPMAS NMR spectrum of I@TAPB-QOT COP; very interestingly, the spectrum obtained was quite different from the one of the unloaded COP (Figure S20). The differences in the spectra were in terms of chemical shifts and linewidths, and they suggest a strong interaction between I_2_ and the COP. Albeit the overlap of the signals and their linewidths makes very hard to properly assign the peaks and characterize the I_2_-loaded COP at an atomic level, one should note that the perturbed chemical shift region (*i.e.*, in the range 135–140 ppm) is the one typical of the C of the thiophene ring in α and β positions with respect to the sulfur,^73^ and a tentative explanation for this would be likely related to the interaction of I_2_ with the sulfur atom, leading to a shielding of the proximal C atoms.

Despite the reliable results obtained by both Raman spectroscopy and calculations, also fairly supported by ^13^C SSNMR, definite information on the charge state of the different atoms could be extracted by XPS; unfortunately, the ultra-high vacuum required would cause the complete leakage of the I_2_ from our material, preventing us to obtain useful information on the absorption mechanism.

**Figure 4:**
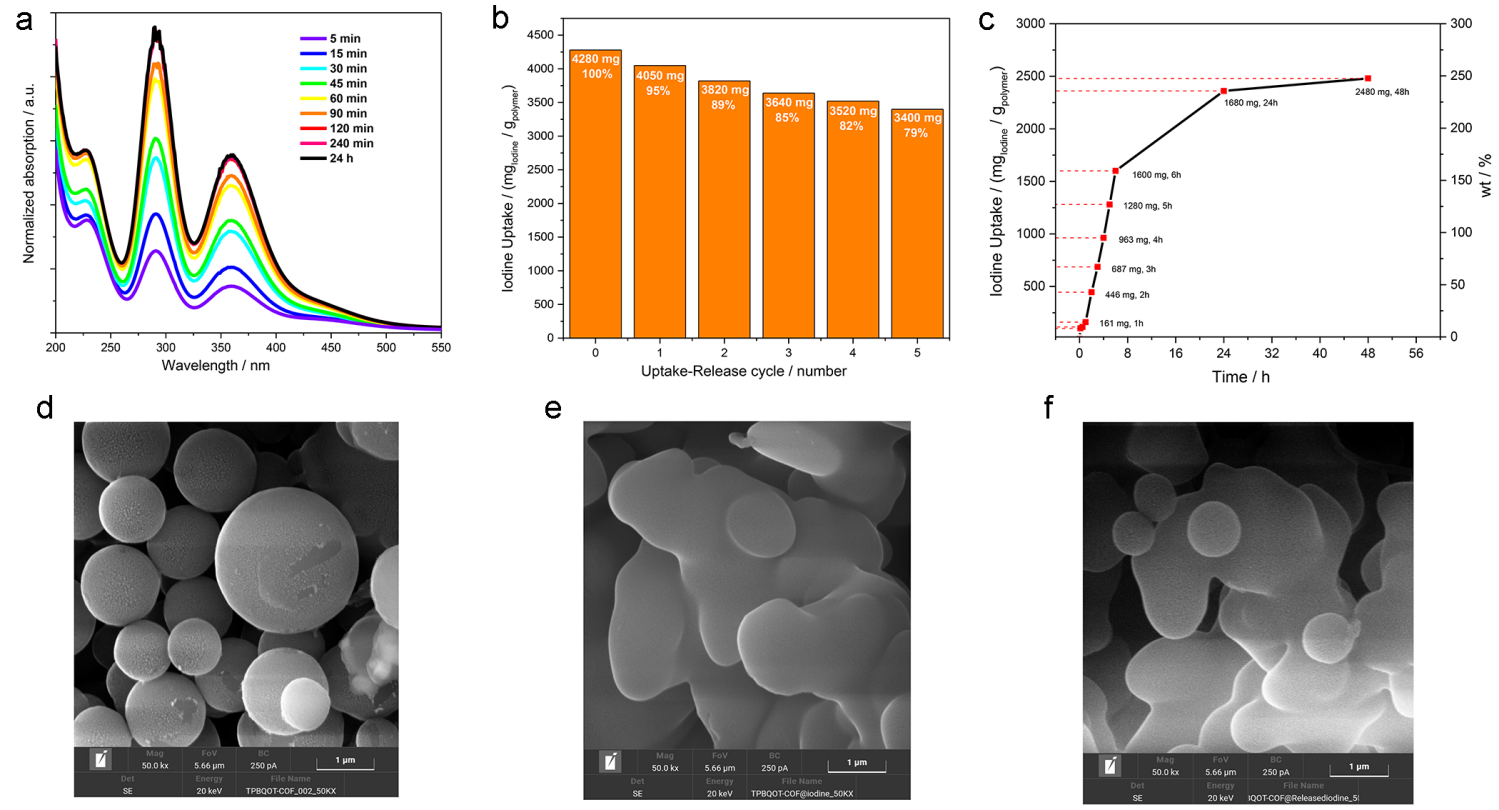
UV–vis spectra at different time of EtOH solution employed for I_2_ release from TBP-QOT-COP (a); amount of I_2_ uptake for each uptake–release cycle for the same TBP-QOT-COP sample (b); gravimetric measurement of iodine uptake (mass polymer = 5 mg; concentration of I_2_ in hexane = 10 mg/mL, volume 5 mL) at 273 K and 1 atm (c); SEM images of TAPB-QOT-COP (d), I@TAPB-QOT-COP (e), and TAPB-QOT-COP-Rel (f). Samples for SEM analyses are the same used for the collection of Raman spectra.

In order to be effectively used as an iodine sponge, besides a good uptake, TAPB-QOT COP has to be proven to also release I_2_ effectively and relatively fast. It should be noted that for radioactive iodine uptake, a chemisorption over sulfur atoms is preferred over a physisorption (*i.e.*, the entrapping of gaseous I_2_ in the pores of the COP), allowing a safer and more controlled uptake/release kinetic. In order to investigate the reversibility of the uptake/release process, we subjected the polymers to multiple cycles. Based on the previously collected data, the uptake process was considered to be completed after 24 h. On the other hand, the release process was conducted by dipping the I@TAPB-QOT-COP in EtOH (Photo S2), and UV–vis spectroscopy was employed to monitor the I_2_ release: the latter was considered complete when the absorption of the solution does not vary with time (*i.e.*, after 120 min, Figure 4a). Indeed, the polymer recyclability was proved to be very good since it retained more than 80% (*i.e.*, 3400 mg g^–1^) of its initial uptake ability after 5 cycles (Figure 4b), comparable or even better than the state-of-the-art references.^45^ The decrement in the I_2_ uptake of TAPB-QOT-COP during cycles could be related to (partial) irreversible chemisorption of molecular species as well as to sequestration of the latter (and possibly the washing solvent) in the inner pores of the materials.^74^ Additionally, as confirmed by both FESEM images and Raman spectra, a slight deformation of the TAPB-QOT-COP’s structure could not be completely ruled out (Figure 4d–f): the latter could reduce the iodine accessibility to some sites.^75^

Finally, we also investigated I_2_ uptake when the molecule is dispersed in an organic solvent (*n*-hexane). Indeed, the solubilization of the gaseous iodine into a solvent followed by its capture by molecular sponges could be a practical approach to limit the leakage of highly volatile I_2_. A fixed amount of iodine (20 mg) was dissolved into hexane (2 mL) and then the TAPB-QOT polymer was added (5 mg). UV–vis spectroscopy was exploited to monitor the uptake reaction by the decrease in the intensity of the characteristic I_2_ peak (at 522 nm, Figure S21). After 48 h, the spectrum remained constant, and an iodine uptake up to 2480 mg/g (248 wt %) was measured (Figure 4c). This indicates that the TAPB-QOT polymer still exhibits a relatively high iodine adsorption capacity even when it is solubilized in hexane. A lower adsorption capacity in solution than in the vapor phase can be due to the impact of the solvent encapsulation.^37^ Indeed, the solvent would (i) compete with iodine to be adsorbed onto the polymer surface and (ii) interact with the I_2_, leading to a higher hydrodynamic radius, preventing the interaction with inner (and smaller) cavities. One should note that, in the present context, our main aim was to prove the effectiveness of the synthetized COP as an I_2_ sponge and its maximum capability for both gaseous and solubilized iodine. Indeed, it would also be very interesting to test the I_2_ uptake when the latter is present in very low concentration (*i.e.*, traces or ppm), but this will require dedicated analyses (also varying the temperature or the solvent nature) that will be discussed in a forthcoming paper.

## 4 Conclusions

Throughout this paper, we designed and synthetized a novel imine-linked 2D covalent organic polymer (COP) containing quinoid oligo thiophene (QOT) and tris(aminophenyl) benzene (TAPB) moieties. Some detailed DFT-based calculations were run to clarify the energetics between the different isomers formed by QOT in reaction conditions, evidencing that isomer C was the most suitable one for the COP formation as a result of both energetic and steric constraints. The synthetized QOT-TAPB-COP showed only poor crystallinity (due to a not-perfect stacking between the 2D layers) and very low surface area (10 or 113 m^2^ g^–1^ when calculated by N_2_ or CO_2_ absorption, respectively), microporosity (pore diameter <8 Å), and low total pore volume (0.018 cm^3^ g^–1^, calculated from CO_2_ absorption). Notwithstanding this, the COP was tested as an effective iodine sponge exhibiting a maximum uptake capacity as high as 464 wt %. This remarkably high value, one of the highest ever reported for a not crystalline and low porous COP, was mainly related to the thoughtfully designed QOT building block; the latter, once implemented in the polymeric structure is highly appropriate for I_2_ uptake, mainly due to its high sulfur content. The pivotal role of S atoms (especially when present in the quinoid form of thiophene) in the I_2_ uptake was further confirmed by Raman spectroscopy and DFT calculations from which a very strong S-I_2_ interaction was evidenced; moreover, EDS mapping showed a superimposing of the S and I distribution and a relative ratio remarkably close to 0.5 (*i.e.*, one sulfur atom for each iodine molecule). Furthermore, excellent recyclability of the QOT-TAPB-COP polymer was proven (higher than 80% after 5 cycles). Our findings are quite exciting, considering the relatively low surface area and porosity of the synthetized materials. The remarkable uptake capacity (464 wt %), together with the relatively low active surface area (113 m^2^ g^–1^), leads to the highest volumetric uptake of iodine ever reported (*i.e.*, 331 g cm^–3^, see Table 1), evidencing how a rational chemical design of the COP’s moieties could largely counterbalance a low surface area and limited pore volume.

## Supporting Information Available

Scheme of the synthesis; photographs indicating the absorption and release of iodine; ^1^H, ^13^C, and ^15^N NMR spectra; geometry of DFT-optimized isomers and COF structural unit with pore diameters; thermogravimetric analyses, adsorption/desorption isotherms; Raman and UV–vis spectra; theoretically predicted infrared vibrational spectra; calculated binding energies; and experimental details (PDF)

## References

[ref1] Vogel J.; Steinberger J. K.; O’Neill D. W.; Lamb W. F.; Krishnakumar J. Socio-Economic Conditions for Satisfying Human Needs at Low Energy Use: An International Analysis of Social Provisioning. Globel Environ. Change 2021, 69, 102287 10.1016/j.gloenvcha.2021.102287.

[ref2] European Commission. REPowerEU Plan.

[ref3] Mushkacheva G.; Rabinovich E.; Privalov V.; Povolotskaya S.; Shorokhova V.; Sokolova S.; Turdakova V.; Ryzhova E.; Hall P.; Schneider A. B.; et al. Thyroid Abnormalities Associated with Protracted Childhood Exposure to ^131^I from Atmospheric Emissions from the Mayak Weapons Facility in Russia. Radiat. Res. 2006, 166, 715–722. 10.1667/rr0410.1. 17067203

[ref4] Lu J. Y. Crystal Engineering of Cu-Containing Metal–Organic Coordination Polymers under Hydrothermal Conditions. Coord. Chem. Rev. 2003, 246, 327–347. 10.1016/j.cct.2003.08.005.

[ref5] Ten Hoeve J. E.; Jacobson M. Z. Worldwide Health Effects of the Fukushima Daiichi Nuclear Accident. Energy Environ. Sci. 2012, 5, 8743–8757. 10.1039/c2ee22019a.

[ref6] Liu S.; Wang N.; Zhang Y.; Li Y.; Han Z.; Na P. Efficient Removal of Radioactive Iodide Ions from Water by Three-Dimensional Ag2O–Ag/TiO2 Composites under Visible Light Irradiation. J. Hazard. Mater. 2015, 284, 171–181. 10.1016/j.jhazmat.2014.10.054. 25463231

[ref7] Subrahmanyam K. S.; Malliakas C. D.; Sarma D.; Armatas G. S.; Wu J.; Kanatzidis M. G. Ion-Exchangeable Molybdenum Sulfide Porous Chalcogel: Gas Adsorption and Capture of Iodine and Mercury. J. Am. Chem. Soc. 2015, 137, 13943–13948. 10.1021/jacs.5b09110. 26456071

[ref8] Deitz V. R. Interaction of Radioactive Iodine Gaseous Species with Nuclear-Grade Activated Carbons. Carbon 1987, 25, 31–38. 10.1016/0008-6223(87)90037-6.

[ref9] Pham T. C. T.; Docao S.; Hwang I. C.; Song M. K.; Choi D. Y.; Moon D.; Oleynikov P.; Yoon K. B. Capture of Iodine and Organic Iodides Using Silica Zeolites and the Semiconductor Behaviour of Iodine in a Silica Zeolite. Energy Environ. Sci. 2016, 9, 1050–1062. 10.1039/c5ee02843d.

[ref10] Chapman K. W.; Chupas P. J.; Nenoff T. M. Radioactive Iodine Capture in Silver-Containing Mordenites through Nanoscale Silver Iodide Formation. J. Am. Chem. Soc. 2010, 132, 8897–8899. 10.1021/ja103110y. 20550110

[ref11] Geng T.; Zhu Z.; Zhang W.; Wang Y. A Nitrogen-Rich Fluorescent Conjugated Microporous Polymer with Triazine and Triphenylamine Units for High Iodine Capture and Nitro Aromatic Compound Detection. J. Mater. Chem. A 2017, 5, 7612–7617. 10.1039/c7ta00590c.

[ref12] Qian X.; Zhu Z.-Q.; Sun H.-X.; Ren F.; Mu P.; Liang W.; Chen L.; Li A. Capture and Reversible Storage of Volatile Iodine by Novel Conjugated Microporous Polymers Containing Thiophene Units. ACS Appl. Mater. Interfaces 2016, 8, 21063–21069. 10.1021/acsami.6b06569. 27458782

[ref13] Riley B. J.; Vienna J. D.; Strachan D. M.; McCloy J. S.; Jerden J. L. Materials and Processes for the Effective Capture and Immobilization of Radioiodine: A Review. J. Nucl. Mater. 2016, 470, 307–326. 10.1016/j.jnucmat.2015.11.038.

[ref14] Côté A. P.; Benin A. I.; Ockwig N. W.; O’Keeffe M.; Matzger A. J.; Yaghi O. M. Porous, Crystalline, Covalent Organic Frameworks. Science 2005, 310, 1166–1170. 10.1126/science.1120411. 16293756

[ref15] Babarao R.; Jiang J. Exceptionally High CO2storage in Covalent-Organic Frameworks: Atomistic Simulation Study. Energy Environ. Sci. 2008, 1, 139–143. 10.1039/b805473h.

[ref16] Wang Z.; Zhang S.; Chen Y.; Zhang Z.; Ma S. Covalent Organic Frameworks for Separation Applications. Chem. Soc. Rev. 2020, 49, 708–735. 10.1039/c9cs00827f. 31993598

[ref17] Yusran Y.; Li H.; Guan X.; Fang Q.; Qiu S. Covalent Organic Frameworks for Catalysis. EnergyChem 2020, 2, 100035 10.1016/j.enchem.2020.100035.

[ref18] Liu X.; Huang D.; Lai C.; Zeng G.; Qin L.; Wang H.; Yi H.; Li B.; Liu S.; Zhang M.; et al. Recent Advances in Covalent Organic Frameworks (COFs) as a Smart Sensing Material. Chem. Soc. Rev. 2019, 48, 5266–5302. 10.1039/c9cs00299e. 31468053

[ref19] Wan S.; Gándara F.; Asano A.; Furukawa H.; Saeki A.; Dey S. K.; Liao L.; Ambrogio M. W.; Botros Y. Y.; Duan X.; et al. Covalent Organic Frameworks with High Charge Carrier Mobility. Chem. Mater. 2011, 23, 4094–4097. 10.1021/cm201140r.

[ref20] Keller N.; Bein T. Optoelectronic Processes in Covalent Organic Frameworks. Chem. Soc. Rev. 2021, 50, 1813–1845. 10.1039/d0cs00793e. 33331358

[ref21] Lin Y. Y.; Song H.; Rao H.; Du Z.; Pan Z.; Zhong X. MOF-Derived Co,N Codoped Carbon/Ti Mesh Counter Electrode for High-Efficiency Quantum Dot Sensitized Solar Cells. J. Phys. Chem. Lett. 2019, 10, 4974–4979. 10.1021/acs.jpclett.9b02082. 31411029

[ref22] Yildirim O.; Bonomo M.; Barbero N.; Atzori C.; Civalleri B.; Bonino F.; Viscardi G.; Barolo C. Application of Metal-Organic Frameworks and Covalent Organic Frameworks as (Photo)Active Material in Hybrid Photovoltaic Technologies. Energies 2020, 13, 5602 10.3390/en13215602.

[ref23] Wu C.; Liu Y.; Liu H.; Duan C.; Pan Q.; Zhu J.; Hu F.; Ma X.; Jiu T.; Li Z.; et al. Highly Conjugated Three-Dimensional Covalent Organic Frameworks Based on Spirobifluorene for Perovskite Solar Cell Enhancement. J. Am. Chem. Soc. 2018, 140, 10016–10024. 10.1021/jacs.8b06291. 30008216

[ref24] Yildirim O.; Derkus B. Triazine-Based 2D Covalent Organic Frameworks Improve the Electrochemical Performance of Enzymatic Biosensors. J. Mater. Sci. 2020, 55, 3034–3044. 10.1007/s10853-019-04254-5.

[ref25] Li J.; Jing X.; Li Q.; Li S.; Gao X.; Feng X.; Wang B. Bulk COFs and COF Nanosheets for Electrochemical Energy Storage and Conversion. Chem. Soc. Rev. 2020, 49, 3565–3604. 10.1039/d0cs00017e. 32369058

[ref26] Bai L.; Gao Q.; Zhao Y. Two Fully Conjugated Covalent Organic Frameworks as Anode Materials for Lithium Ion Batteries. J. Mater. Chem. A 2016, 4, 14106–14110. 10.1039/c6ta06449c.

[ref27] DeBlase C. R.; Silberstein K. E.; Truong T.-T.; Abruña H. D.; Dichtel W. R. β-Ketoenamine-Linked Covalent Organic Frameworks Capable of Pseudocapacitive Energy Storage. J. Am. Chem. Soc. 2013, 135, 16821–16824. 10.1021/ja409421d. 24147596

[ref28] Khattak A. M.; Ghazi Z. A.; Liang B.; Khan N. A.; Iqbal A.; Li L.; Tang Z. A Redox-Active 2D Covalent Organic Framework with Pyridine Moieties Capable of Faradaic Energy Storage. J. Mater. Chem. A 2016, 4, 16312–16317. 10.1039/c6ta05784e.

[ref29] Ding S.-Y.; Gao J.; Wang Q.; Zhang Y.; Song W.-G.; Su C.-Y.; Wang W. Construction of Covalent Organic Framework for Catalysis: Pd/COF-LZU1 in Suzuki–Miyaura Coupling Reaction. J. Am. Chem. Soc. 2011, 133, 19816–19822. 10.1021/ja206846p. 22026454

[ref30] Skorjanc T.; Shetty D.; Trabolsi A. Pollutant Removal with Organic Macrocycle-Based Covalent Organic Polymers and Frameworks. Chem 2021, 7, 882–918. 10.1016/j.chempr.2021.01.002.

[ref31] Furukawa H.; Yaghi O. M. Storage of Hydrogen, Methane, and Carbon Dioxide in Highly Porous Covalent Organic Frameworks for Clean Energy Applications. J. Am. Chem. Soc. 2009, 131, 8875–8883. 10.1021/ja9015765. 19496589

[ref32] Xie W.; Cui D.; Zhang S. R.; Xu Y. H.; Jiang D. L. Iodine Capture in Porous Organic Polymers and Metal-Organic Frameworks Materials. Mater. Horiz. 2019, 6, 1571–1595. 10.1039/c8mh01656a.

[ref33] Pei C.; Ben T.; Xu S.; Qiu S. Ultrahigh Iodine Adsorption in Porous Organic Frameworks. J. Mater. Chem. A 2014, 2, 7179–7187. 10.1039/c4ta00049h.

[ref34] An S.; Zhu X.; He Y.; Yang L.; Wang H.; Jin S.; Hu J.; Liu H. Porosity Modulation in Two-Dimensional Covalent Organic Frameworks Leads to Enhanced Iodine Adsorption Performance. Ind. Eng. Chem. Res. 2019, 58, 10495–10502. 10.1021/acs.iecr.9b00028.

[ref35] Yin Z.-J.; Xu S.-Q.; Zhan T.-G.; Qi Q.-Y.; Wu Z.-Q.; Zhao X. Ultrahigh Volatile Iodine Uptake by Hollow Microspheres Formed from a Heteropore Covalent Organic Framework. Chem. Commun. 2017, 53, 7266–7269. 10.1039/c7cc01045a. 28265612

[ref36] Pan X.; Qin X.; Zhang Q.; Ge Y.; Ke H.; Cheng G. N- and S-Rich Covalent Organic Framework for Highly Efficient Removal of Indigo Carmine and Reversible Iodine Capture. Microporous Mesoporous Mater. 2020, 296, 109990 10.1016/j.micromeso.2019.109990.

[ref37] Du W.; Qin Y.; Ni C.; Dai W.; Zou J. Efficient Capture of Volatile Iodine by Thiophene-Containing Porous Organic Polymers. ACS Appl. Polym. Mater. 2020, 2, 5121–5128. 10.1021/acsapm.0c00907.

[ref38] Lee J.-S. M.; Cooper A. I. Advances in Conjugated Microporous Polymers. Chem. Rev. 2020, 120, 2171–2214. 10.1021/acs.chemrev.9b00399. 31990527 PMC7145355

[ref39] Sun H.; La P.; Yang R.; Zhu Z.; Liang W.; Yang B.; Li A.; Deng W. Innovative Nanoporous Carbons with Ultrahigh Uptakes for Capture and Reversible Storage of CO2 and Volatile Iodine. J. Hazard. Mater. 2017, 321, 210–217. 10.1016/j.jhazmat.2016.09.015. 27619967

[ref40] Liao Y.; Weber J.; Mills B. M.; Ren Z.; Faul C. F. J. Highly Efficient and Reversible Iodine Capture in Hexaphenylbenzene-Based Conjugated Microporous Polymers. Macromolecules 2016, 49, 6322–6333. 10.1021/acs.macromol.6b00901.

[ref41] Li H.; Ding X.; Han B.-H. Porous Azo-Bridged Porphyrin–Phthalocyanine Network with High Iodine Capture Capability. Chem.—Eur. J. 2016, 22, 11863–11868. 10.1002/chem.201602337. 27412919

[ref42] Yan Z.; Yuan Y.; Tian Y.; Zhang D.; Zhu G. Highly Efficient Enrichment of Volatile Iodine by Charged Porous Aromatic Frameworks with Three Sorption Sites. Angew. Chem., Int. Ed. 2015, 54, 12733–12737. 10.1002/anie.201503362. 26316032

[ref43] Das G.; Prakasam T.; Nuryyeva S.; Han D. S.; Abdel-Wahab A.; Olsen J.-C.; Polychronopoulou K.; Platas-Iglesias C.; Ravaux F.; Jouiad M.; et al. Multifunctional Redox-Tuned Viologen-Based Covalent Organic Polymers. J. Mater. Chem. A 2016, 4, 15361–15369. 10.1039/c6ta06439f.

[ref44] Geng T.; Ye S.; Zhu Z.; Zhang W. Triazine-Based Conjugated Microporous Polymers with N,N,N′,N′-Tetraphenyl-1,4-Phenylenediamine, 1,3,5-Tris(Diphenylamino)Benzene and 1,3,5-Tris[(3-Methylphenyl)-Phenylamino]Benzene as the Core for High Iodine Capture and Fluorescence Sensing of o-Nitrophe. J. Mater. Chem. A 2018, 6, 2808–2816. 10.1039/c7ta08251g.

[ref45] Mohan A.; Al-Sayah M. H.; Ahmed A.; El-Kadri O. M. Triazine-Based Porous Organic Polymers for Reversible Capture of Iodine and Utilization in Antibacterial Application. Sci. Rep. 2022, 12, 2638 10.1038/s41598-022-06671-0. 35173259 PMC8850422

[ref46] Lan Y.; Tong M.; Yang Q.; Zhong C. Computational Screening of Covalent Organic Frameworks for the Capture of Radioactive Iodine and Methyl Iodide. CrystEngComm 2017, 19, 4920–4926. 10.1039/c7ce00118e.

[ref47] Chen W. C.; Jenekhe S. A. Model Compound Studies of Small Bandgap Conjugated Poly(Heteroarylene Methines). Macromol. Chem. Phys. 1998, 199, 655–666. 10.1002/(sici)1521-3935(19980401)199:4<655::aid-macp655>3.0.co;2-u.

[ref48] Guo K.; Wu B.; Jiang Y.; Wang Z.; Liang Z.; Li Y.; Deng Y.; Geng Y. Synthesis of an Isomerically Pure Thienoquinoid for Unipolar N-Type Conjugated Polymers: Effect of Backbone Curvature on Charge Transport Performance. J. Mater. Chem. C 2019, 7, 10352–10359. 10.1039/c9tc03556g.

[ref49] Haase F.; Gottschling K.; Stegbauer L.; Germann L. S.; Gutzler R.; Duppel V.; Vyas V. S.; Kern K.; Dinnebier R. E.; Lotsch B. V. Tuning the Stacking Behaviour of a 2D Covalent Organic Framework through Non-Covalent Interactions. Mater. Chem. Front. 2017, 1, 1354–1361. 10.1039/c6qm00378h.

[ref50] Yang J.; Kang F.; Wang X.; Zhang Q. Design Strategies for Improving the Crystallinity of Covalent Organic Frameworks and Conjugated Polymers: A Review. Mater. Horiz. 2022, 9, 121–146. 10.1039/d1mh00809a. 34842260

[ref51] Li X.; Gao Q.; Aneesh J.; Xu H.-S.; Chen Z.; Tang W.; Liu C.; Shi X.; Adarsh K. V.; Lu Y.; et al. Molecular Engineering of Bandgaps in Covalent Organic Frameworks. Chem. Mater. 2018, 30, 5743–5749. 10.1021/acs.chemmater.8b02560.

[ref52] Biswal B. P.; Chandra S.; Kandambeth S.; Lukose B.; Heine T.; Banerjee R. Mechanochemical Synthesis of Chemically Stable Isoreticular Covalent Organic Frameworks. J. Am. Chem. Soc. 2013, 135, 5328–5331. 10.1021/ja4017842. 23521070

[ref53] Thommes M.; Cychosz K. A. Physical Adsorption Characterization of Nanoporous Materials: Progress and Challenges. Adsorption 2014, 20, 233–250. 10.1007/s10450-014-9606-z.

[ref54] Lozano-Castelló D.; Cazorla-Amorós D.; Linares-Solano A. Usefulness of CO2 Adsorption at 273 K for the Characterization of Porous Carbons. Carbon 2004, 42, 1233–1242. 10.1016/j.carbon.2004.01.037.

[ref55] Zhou M.; Li Z.; Munyentwali A.; Li C.; Shui H.; Li H. Highly Conjugated Two-Dimensional Covalent Organic Frameworks for Efficient Iodine Uptake. Chem. Asian J. 2022, 17, e202200358 10.1002/asia.202200358. 35607250

[ref56] Wang P.; Xu Q.; Li Z.; Jiang W.; Jiang Q.; Jiang D. Exceptional Iodine Capture in 2D Covalent Organic Frameworks. Adv. Mater. 2018, 30, 1801991 10.1002/adma.201801991. 29806216

[ref57] Xu M.; Wang T.; Zhou L.; Hua D. Fluorescent Conjugated Mesoporous Polymers with N,N-Diethylpropylamine for the Efficient Capture and Real-Time Detection of Volatile Iodine. J. Mater. Chem. A 2020, 8, 1966–1974. 10.1039/c9ta11446g.

[ref58] Su K.; Wang W.; Li B.; Yuan D. Azo-Bridged Calix[4]Resorcinarene-Based Porous Organic Frameworks with Highly Efficient Enrichment of Volatile Iodine. ACS Sustain. Chem. Eng. 2018, 6, 17402–17409. 10.1021/acssuschemeng.8b05203.

[ref59] Guo Z.; Sun P.; Zhang X.; Lin J.; Shi T.; Liu S.; Sun A.; Li Z. Amorphous Porous Organic Polymers Based on Schiff-Base Chemistry for Highly Efficient Iodine Capture. Chem. Asian J. 2018, 13, 2046–2053. 10.1002/asia.201800698. 29873203

[ref60] Qian X.; Wang B.; Zhu Z.-Q.; Sun H.-X.; Ren F.; Mu P.; Ma C.; Liang W.-D.; Li A. Novel N-Rich Porous Organic Polymers with Extremely High Uptake for Capture and Reversible Storage of Volatile Iodine. J. Hazard. Mater. 2017, 338, 224–232. 10.1016/j.jhazmat.2017.05.041. 28570876

[ref61] Ren F.; Zhu Z.; Qian X.; Liang W.; Mu P.; Sun H.; Liu J.; Li A. Novel Thiophene-Bearing Conjugated Microporous Polymer Honeycomb-like Porous Spheres with Ultrahigh Iodine Uptake. Chem. Commun. 2016, 52, 9797–9800. 10.1039/c6cc05188j. 27417941

[ref62] Weng J.-Y.; Xu Y.-L.; Song W.-C.; Zhang Y.-H. Tuning the Adsorption and Fluorescence Properties of Aminal-Linked Porous Organic Polymers through N-Heterocyclic Group Decoration. J. Polym. Sci., Part A: Polym. Chem. 2016, 54, 1724–1730. 10.1002/pola.28028.

[ref63] Dai D.; Yang J.; Zou Y. C.; Wu J. R.; Tan L. L.; Wang Y.; Li B.; Lu T.; Wang B.; Yang Y. W. Macrocyclic Arenes-Based Conjugated Macrocycle Polymers for Highly Selective CO2 Capture and Iodine Adsorption. Angew. Chem., Int. Ed. 2021, 60, 8967–8975. 10.1002/anie.202015162. 33539618

[ref64] Hulkko E.; Kiljunen T.; Kiviniemi T.; Pettersson M. From Monomer to Bulk: Appearance of the Structural Motif of Solid Iodine in Small Clusters. J. Am. Chem. Soc. 2009, 131, 1050–1056. 10.1021/ja806537u. 19123809

[ref65] Usseglio S.; Damin A.; Scarano D.; Bordiga S.; Zecchina A.; Lamberti C. (I2)n Encapsulation inside TiO2: A Way to Tune Photoactivity in the Visible Region. J. Am. Chem. Soc. 2007, 129, 2822–2828. 10.1021/ja066083m. 17305337

[ref66] Xie Y.; Pan T.; Lei Q.; Chen C.; Dong X.; Yuan Y.; Shen J.; Cai Y.; Zhou C.; Pinnau I.; et al. Ionic Functionalization of Multivariate Covalent Organic Frameworks to Achieve an Exceptionally High Iodine-Capture Capacity. Angew. Chem., Int. Ed. 2021, 60, 22432–22440. 10.1002/anie.202108522. 34431190

[ref67] Song S.; Shi Y.; Liu N.; Liu F. C-N Linked Covalent Organic Framework for the Efficient Adsorption of Iodine in Vapor and Solution. RSC Adv. 2021, 11, 10512–10523. 10.1039/d0ra10587b. 35423582 PMC8695655

[ref68] Sun Y.; Song S.; Xiao D.; Gan L.; Wang Y. Easily Constructed Imine-Bonded COFs for Iodine Capture at Ambient Temperature. ACS Omega 2020, 5, 24262–24271. 10.1021/acsomega.0c02382. 33015443 PMC7528167

[ref69] Deplano P.; Ferraro J. R.; Mercuri M. L.; Trogu E. F. Structural and Raman Spectroscopic Studies as Complementary Tools in Elucidating the Nature of the Bonding in Polyiodides and in Donor-I2 Adducts. Coord. Chem. Rev. 1999, 188, 71–95. 10.1016/s0010-8545(98)00238-0.

[ref70] Weng K.-F.; Shi Y.; Zheng X.; Phillips D. L. Resonance Raman Investigation of the Short-Time Photodissociation Dynamics of the Charge-Transfer Absorption of the I 2 −Benzene Complex in Benzene Solution. J. Phys. Chem. A 2006, 110, 851–860. 10.1021/jp055069d. 16419981

[ref71] Kiviniemi T.; Hulkko E.; Kiljunen T.; Pettersson M. Iodine–Benzene Complex as a Candidate for a Real-Time Control of a Bimolecular Reaction. Spectroscopic Studies of the Properties of the 1:1 Complex Isolated in Solid Krypton. J. Phys. Chem. A 2009, 113, 6326–6333. 10.1021/jp902012u. 19425545

[ref72] Widdifield C. M.; Bryce D. L. Solid-State 127 I NMR and GIPAW DFT Study of Metal Iodides and Their Hydrates: Structure, Symmetry, and Higher-Order Quadrupole-Induced Effects. J. Phys. Chem. A 2010, 114, 10810–10823. 10.1021/jp108237x. 20860347

[ref73] Reddy M. K.; Varathan E.; Lobo N. P.; Das B. B.; Narasimhaswamy T.; Ramanathan K. V. High-Resolution Solid State 13 C NMR Studies of Bent-Core Mesogens of Benzene and Thiophene. J. Phys. Chem. C 2014, 118, 15044–15053. 10.1021/jp504835c.

[ref74] Raman V. I.; Palmese G. R. Design and Characterization of Nanoporous Polymeric Materials via Reactive Encapsulation of a Chemically Inert Solvent. Colloids Surf., A 2004, 241, 119–125. 10.1016/j.colsurfa.2004.04.032.

[ref75] Wang C.; Wang Y.; Ge R.; Song X.; Xing X.; Jiang Q.; Lu H.; Hao C.; Guo X.; Gao Y.; et al. A 3D Covalent Organic Framework with Exceptionally High Iodine Capture Capability. Chem.—Eur. J. 2018, 24, 585–589. 10.1002/chem.201705405. 29178592

