# Supplementary material for: Quinoid-Thiophene-Based Covalent Organic Polymers for High Iodine Uptake: When Rational Chemical Design Counterbalances the Low Surface Area and Pore Volume
Source: ACS Appl Mater Interfaces. 2023 Mar 16;15(12):15819–31. doi: 10.1021/acsami.2c20853 (PMC10064318; doi:10.1021/acsami.2c20853)
Supplement: Supplementary file 1 — am2c20853_si_001.pdf [file am2c20853_si_001.pdf]

Supporting Information

Quinoid-thiophene based Covalent Organic Polymers for High Iodine Uptake: When Rational Chemical Design Counterbalances Low Surface Area and Pores Volume

Onur Yildirim[a], Arshak Tsaturyan[a,b,c], Alessandro Damin[a,d], Stefano Nejrotti[a,d], Valentina Crocellà[a,d], Angelo Gallo[a], Michele Remo Chierotti[a], Matteo Bonomo[,a,d], Claudia Barolo[a,d,e]*

[a] Department of Chemistry and NIS Interdepartmental Centre, University of Turin, Via Pietro Giuria 7, 10125, Torino, Italy;

[b] Institute of Physical and Organic Chemistry, Southern Federal University, Rostov-on-Don, Russia

[c] Univ Lyon, UJM-Saint-Etienne, CNRS, IOGS, Laboratoire Hubert Curien UMR5516, F-42023 St-Etienne, France

[d] INSTM Reference Centre, Università degli Studi di Torino, Via Gioacchino Quarello 15/a, 10125 Torino, Italy

[e] ICxT Interdepartmental Centre, Università degli Studi di Torino, Via Lungo Dora Siena 100, 10153 Torino, Italy

email: matteo.bonomo@unito.it

Supporting Information

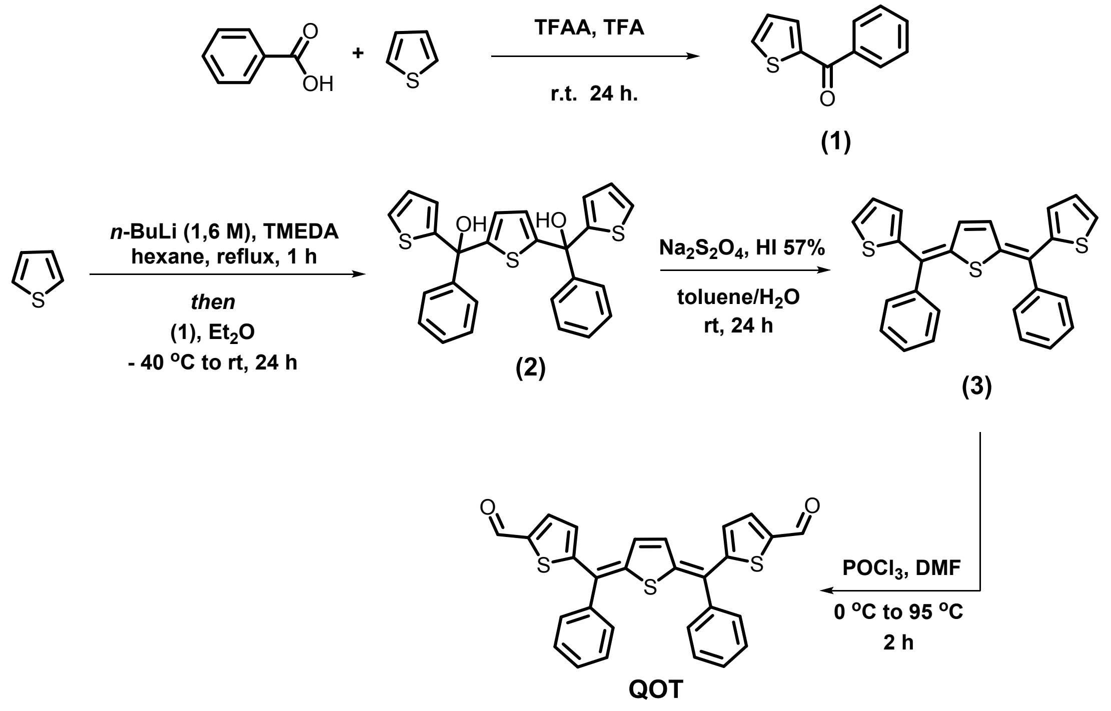

Scheme S1 *Synthesis of thiophene-based quinoid-oligothiophene containing phenyl ring on methine bridge.*

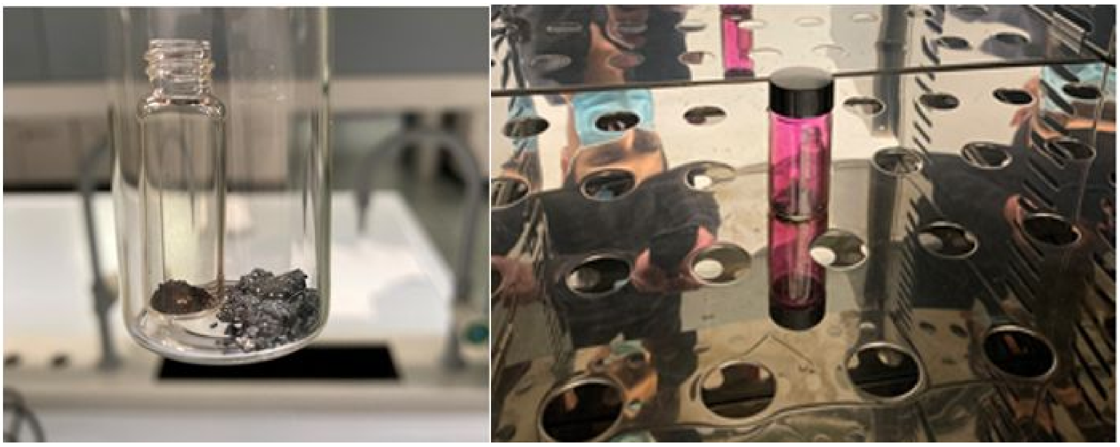

Photo S1 *Photograph of small vial filled with the polymer which is inside of the large vial filled with iodine pieces (on the left) and a vial after iodine adsorption process in the oven (on the right)*

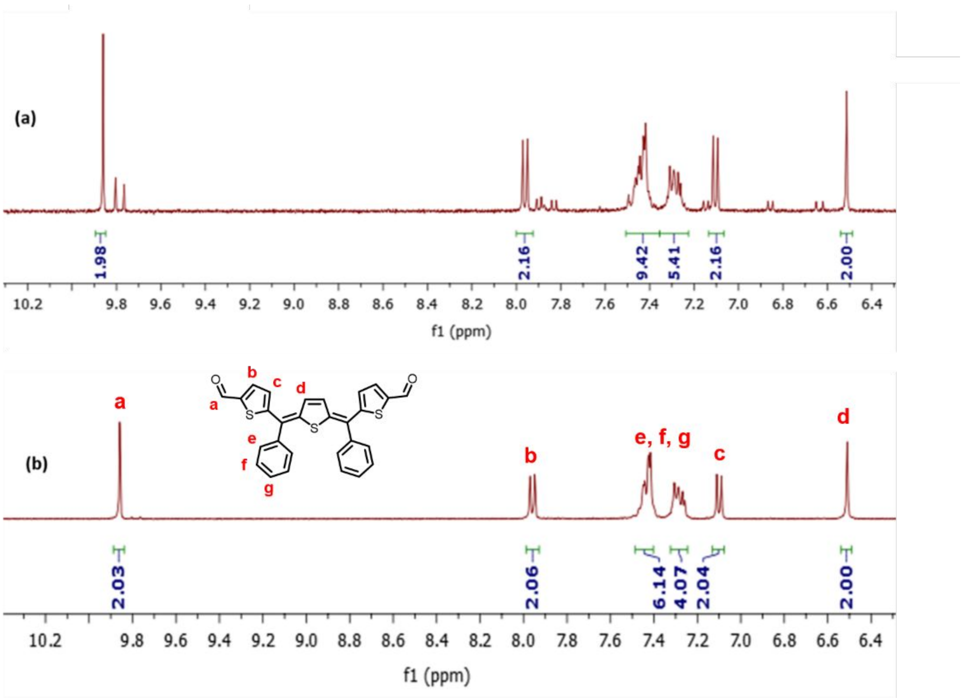

***Figure S1**. ^{1}H-NMR of QOT (a) crude (b) isolated*

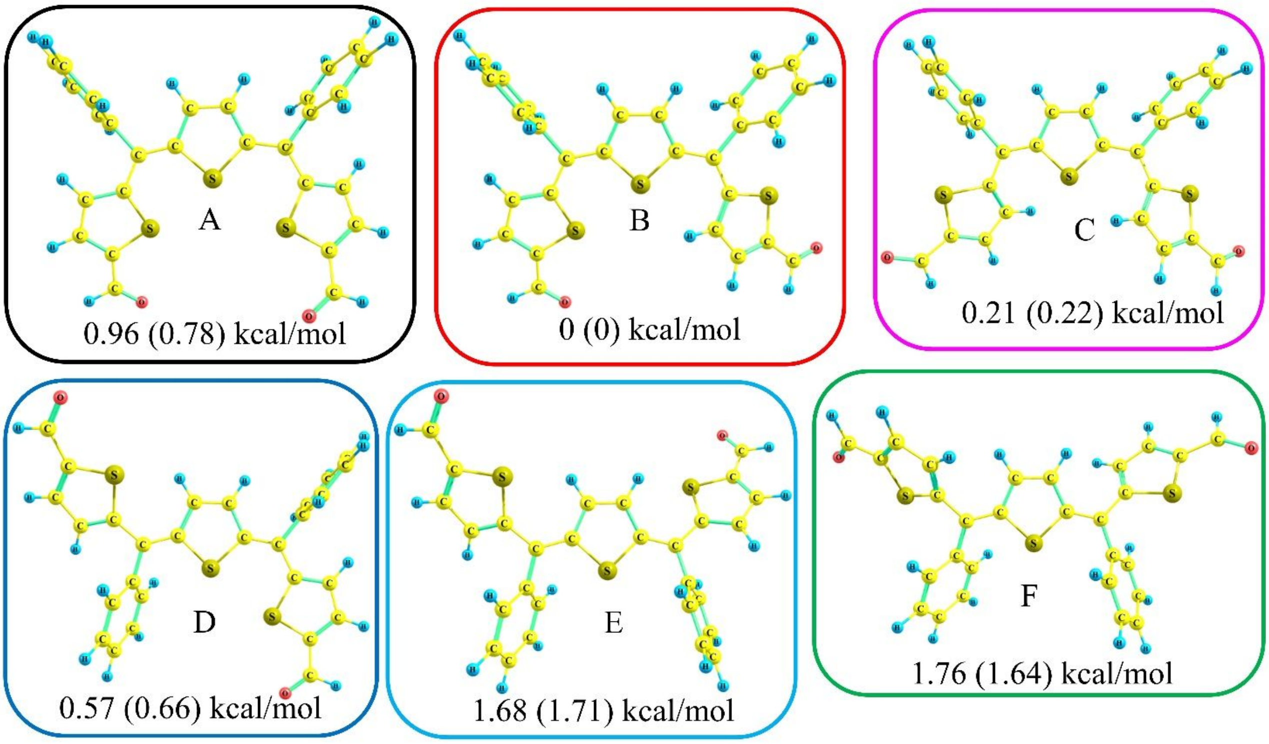

***Figure S2**. Geometry of optimized isomers of the QOT building block. The relative energy in the gas phase and the dioxane solution (in the branches) were shown.*

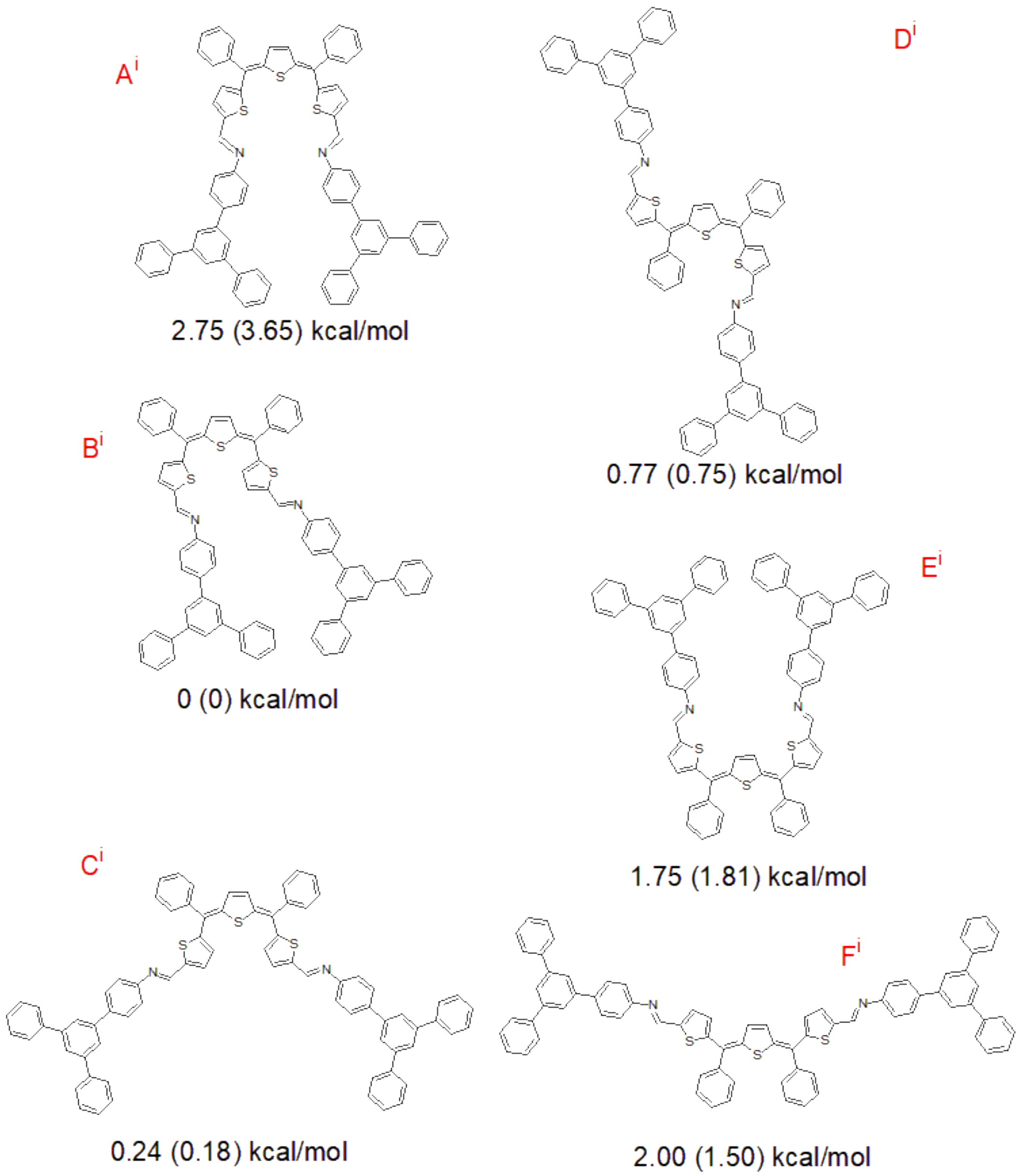

Figure S3. *Model compounds of QOT containing isomers. The relative energy in gas phase and in the dioxane solution (in the branches) were shown*

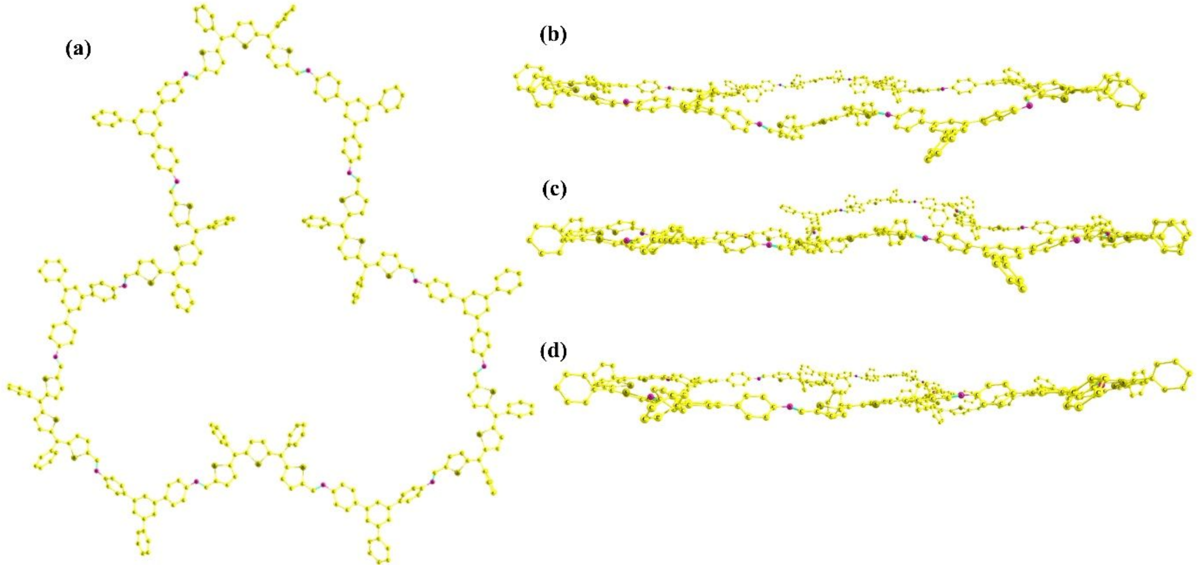

Figure S4. *Top view (a) and side views (b, c, d) of optimized structure of COF structural unit. The Hydrogen atoms are omitted for clarity.*

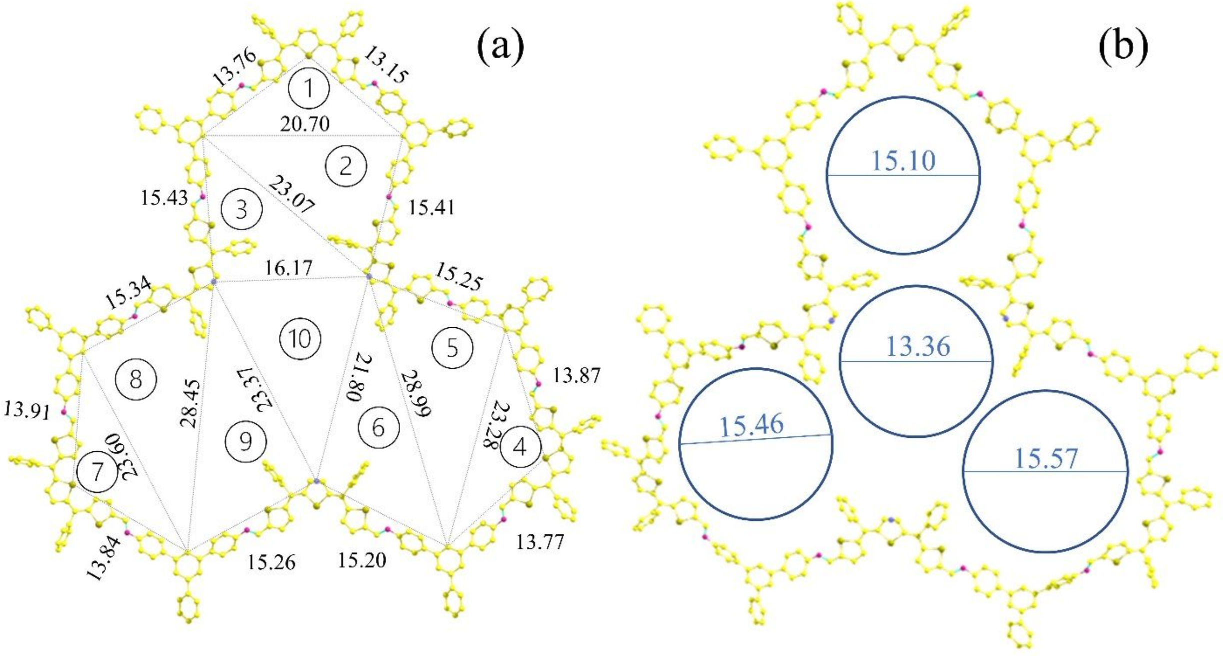

Figure S5. *Optimized structure of the COF unit with inscribed polygon based on 10 triangles (Å) (a) and pore diameters (Å) (b). The length of the side of triangles and interatomic distances in A were shown. The Hydrogen atoms are omitted for clarity.*

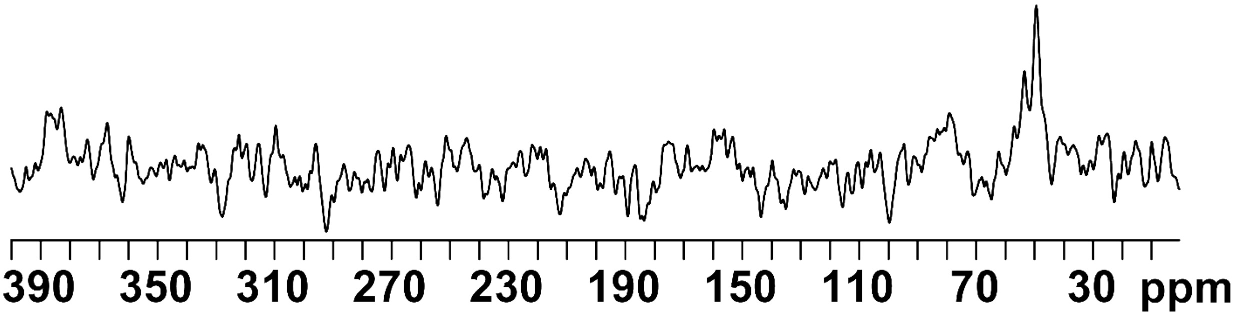

Figure S6. 1D 15 N (60.82 MHz) CPMAS spectrum of TAPB sample acquired with a spinning speed of 12 kHz.

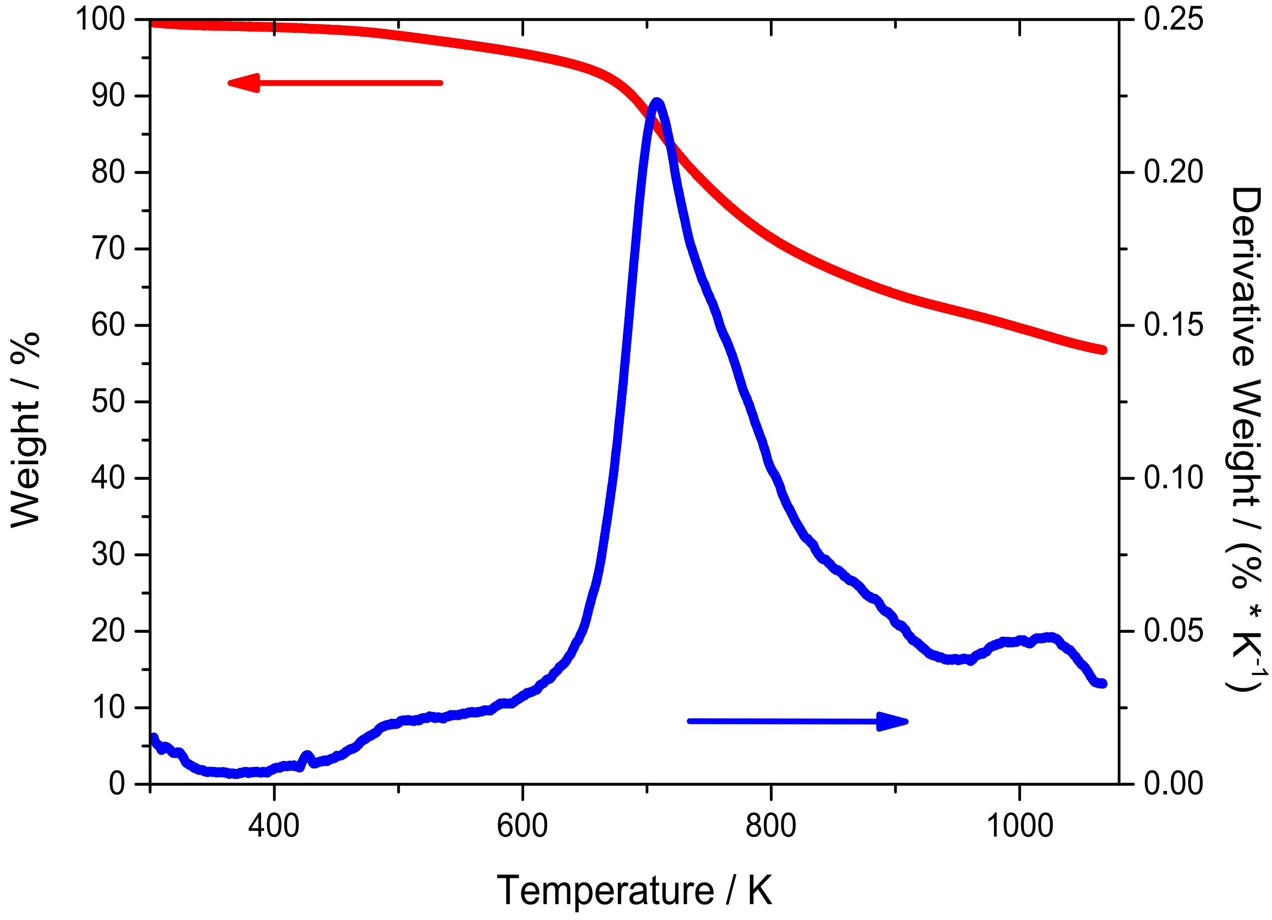

Figure S7. *Thermogravimetric Analyses (in red) and Derivative of TA (in black) of TPB-QOT COP under Nitrogen flux. Dashed line represent the Temperature at which the Material loses 5% in weight.*

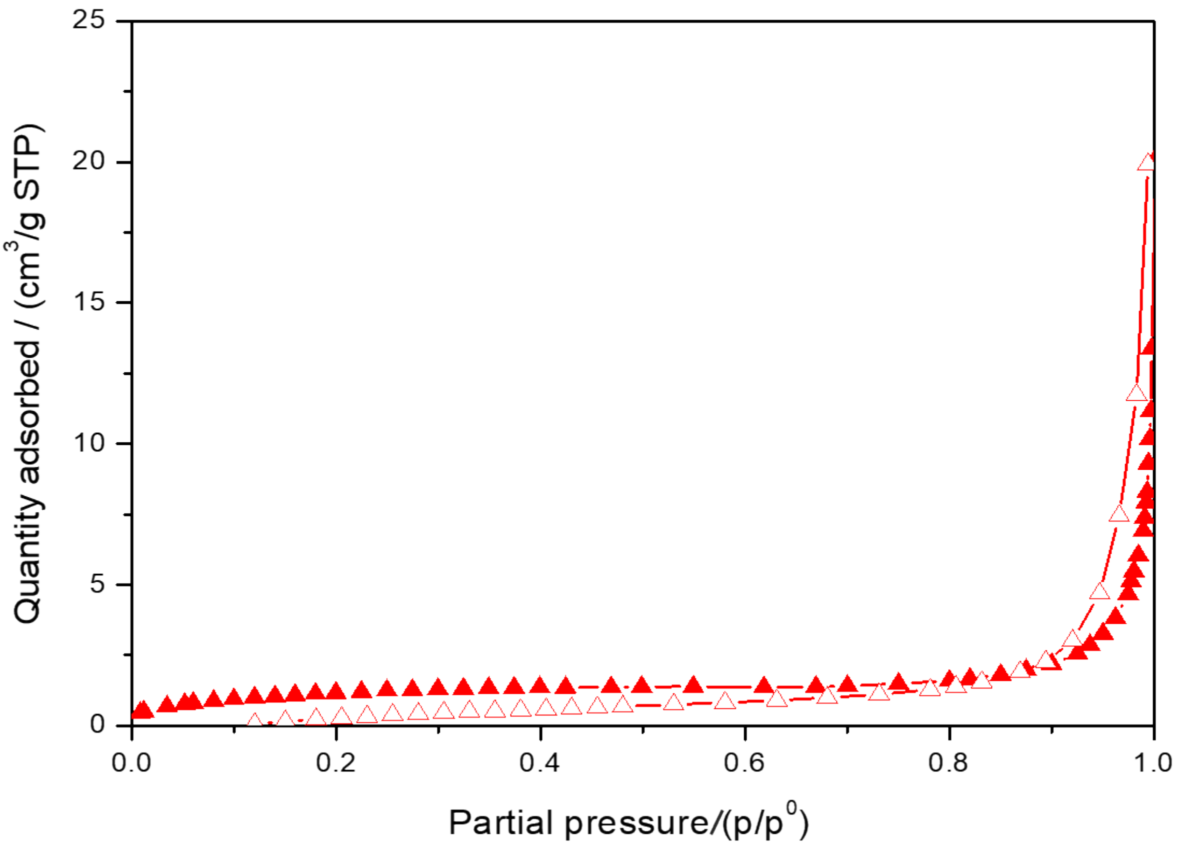

Figure S8. *N_2 adsorption/desorption isotherms collected a 78 K on TPB-QOT COP. Full and empty symbols refer to the adsorption and desorption branch respectively.*

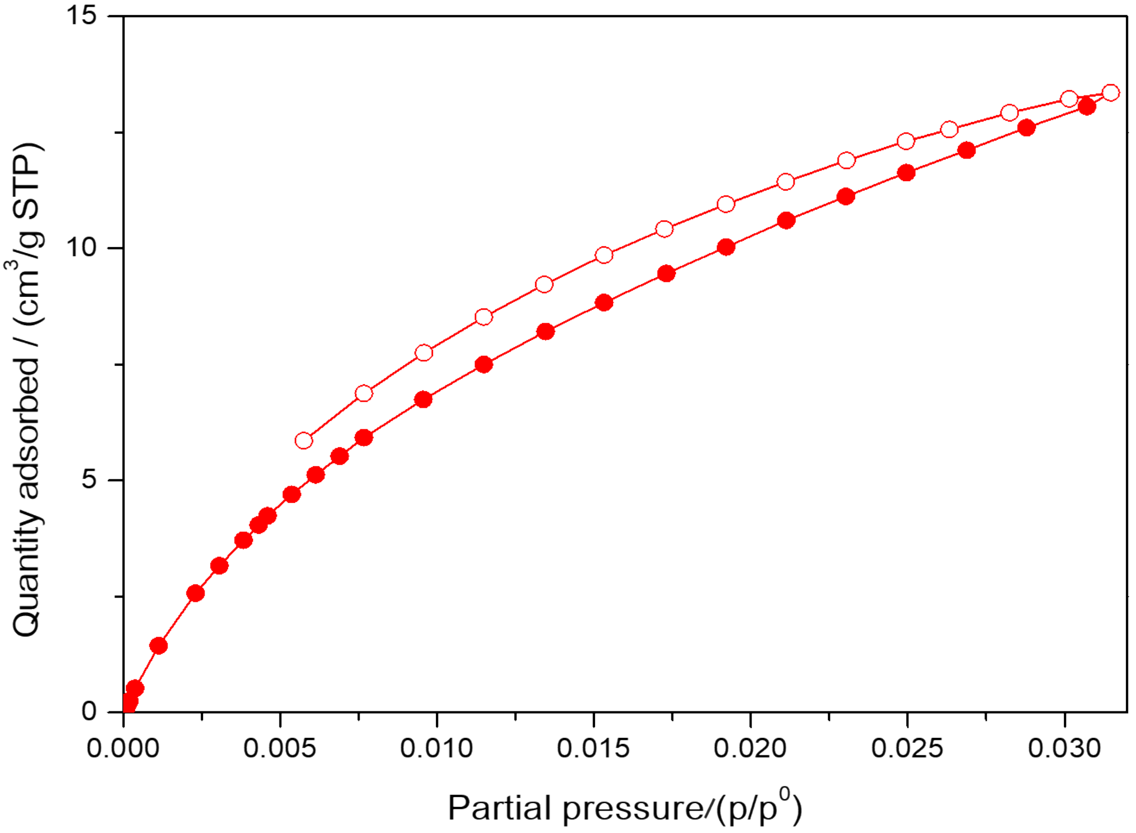

Figure S9. *CO_2 adsorption/desorption isotherms collected at 273 K on TPB-QOT COP. Full and empty symbols refer to the adsorption and desorption branch respectively.*

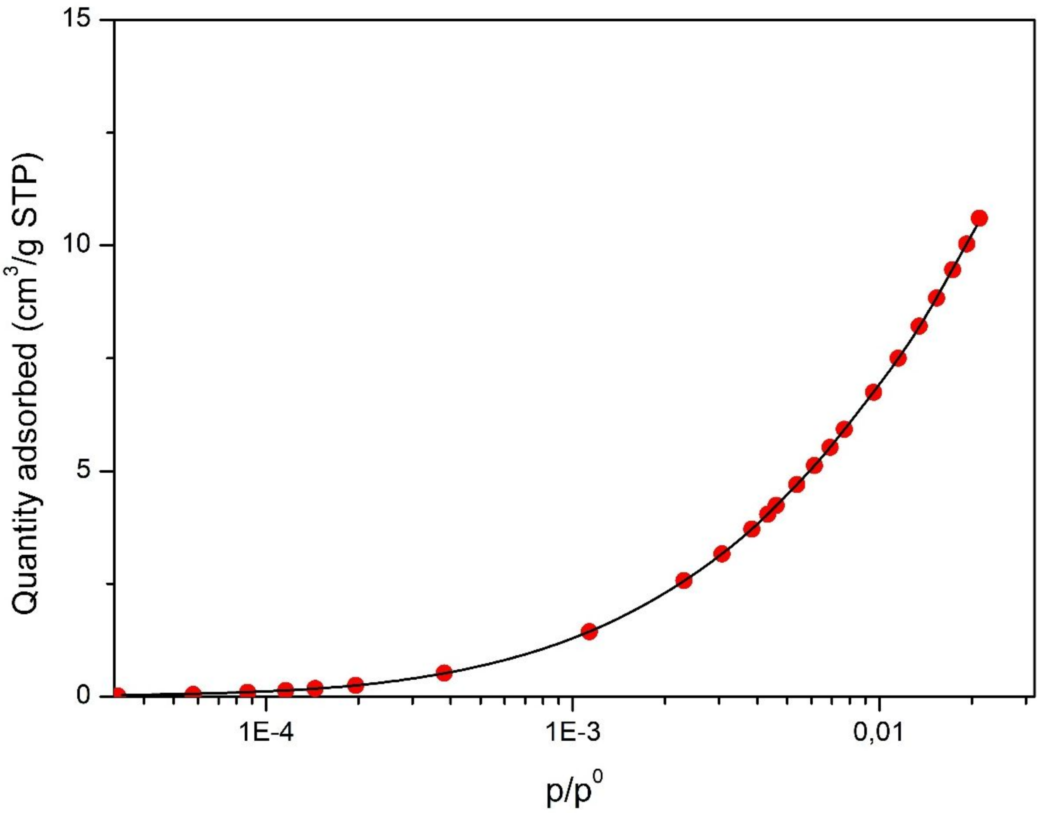

Figure S10. *NL-DFT Pore Size Log Goodness of Fit Graph obtained by applying the NL-DFT analysis to the CO_2 adsorption isotherm collected at 273 K, employing a slit pore geometry and applying a model for CO_2 adsorption at 273 K on carbons.*

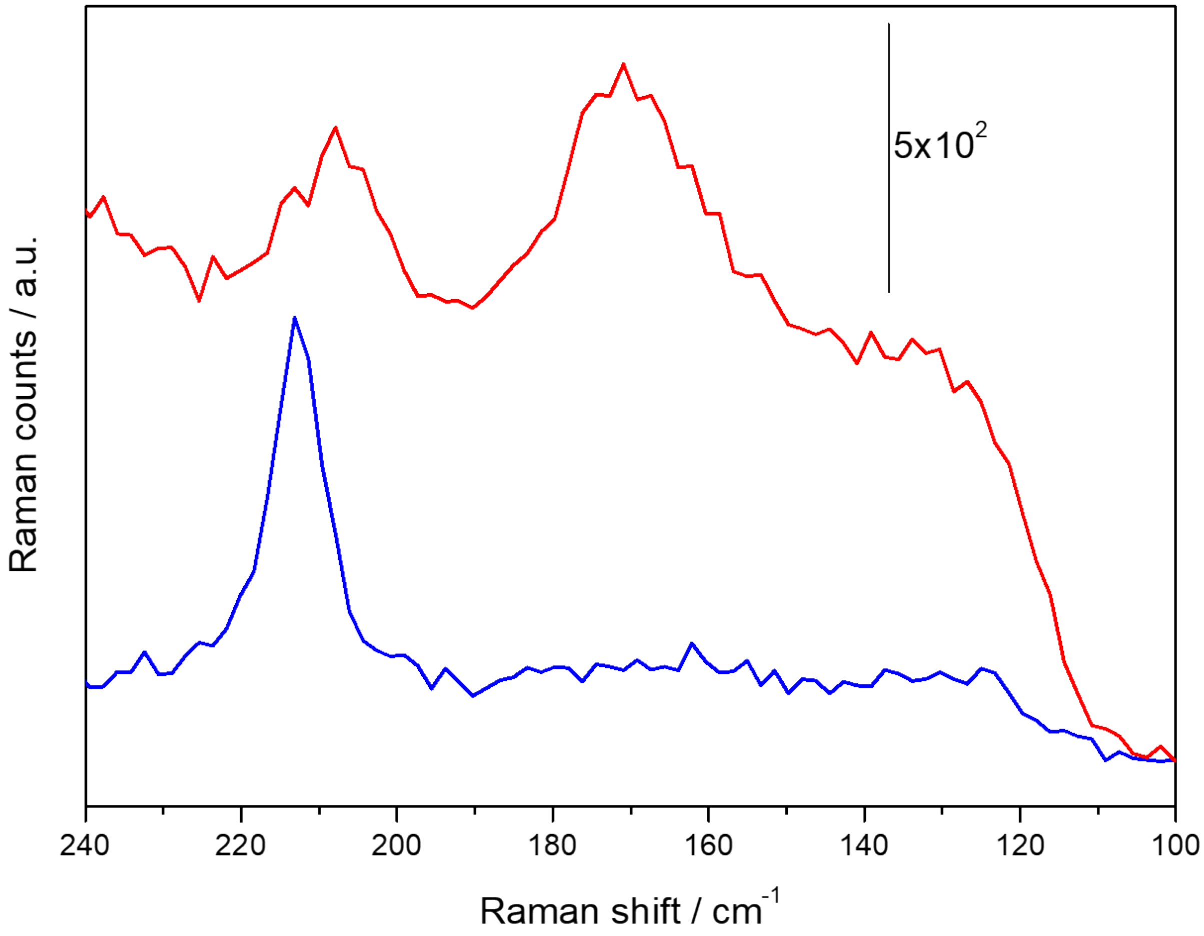

Figure S11. *Raman Spectra of I_2 solution of hexane (in blu) and of the same solution after the addition of 3-hexylthiophene (in red).*

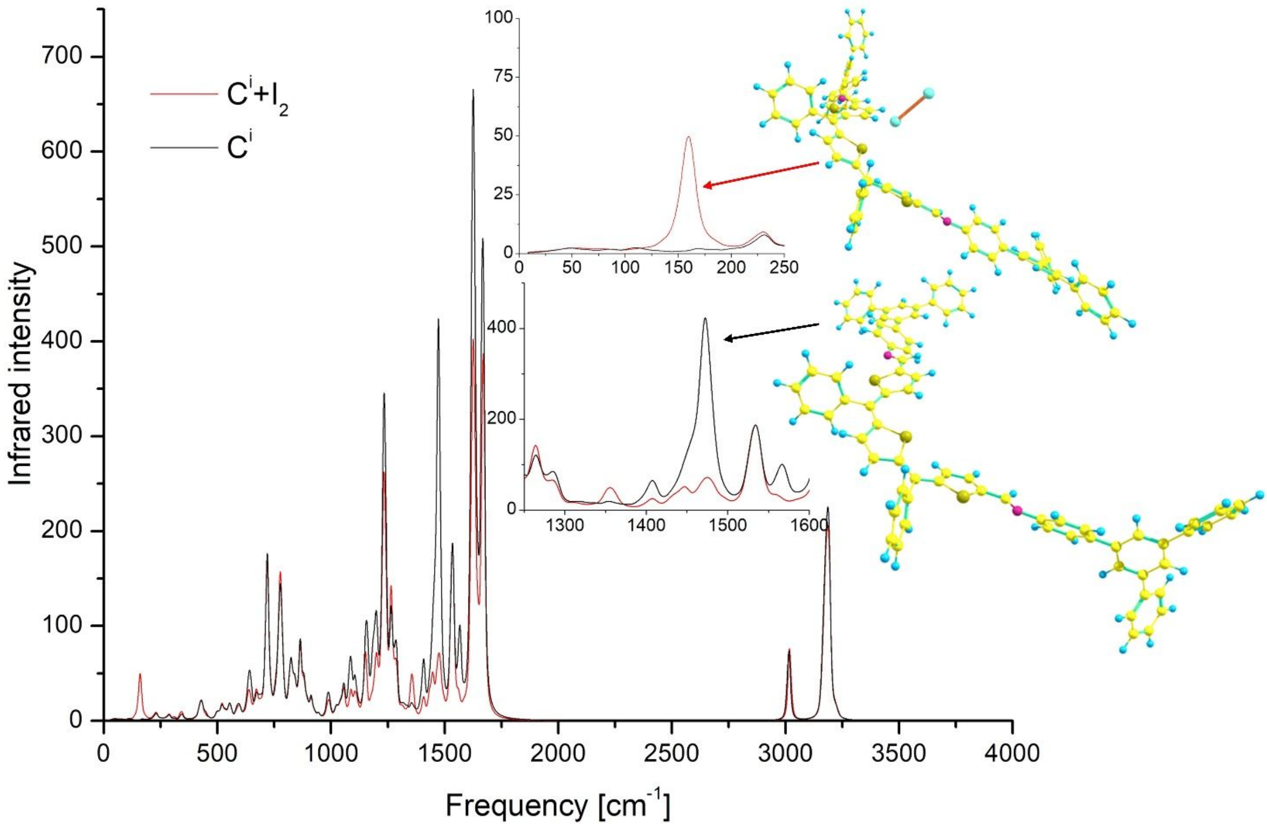

Figure S12. *Calculated Infrared vibrational spectra of isomer C^{i} with two zoom regions from 0 to 250 and 1200 to 1600 cm^{-1} insert.*

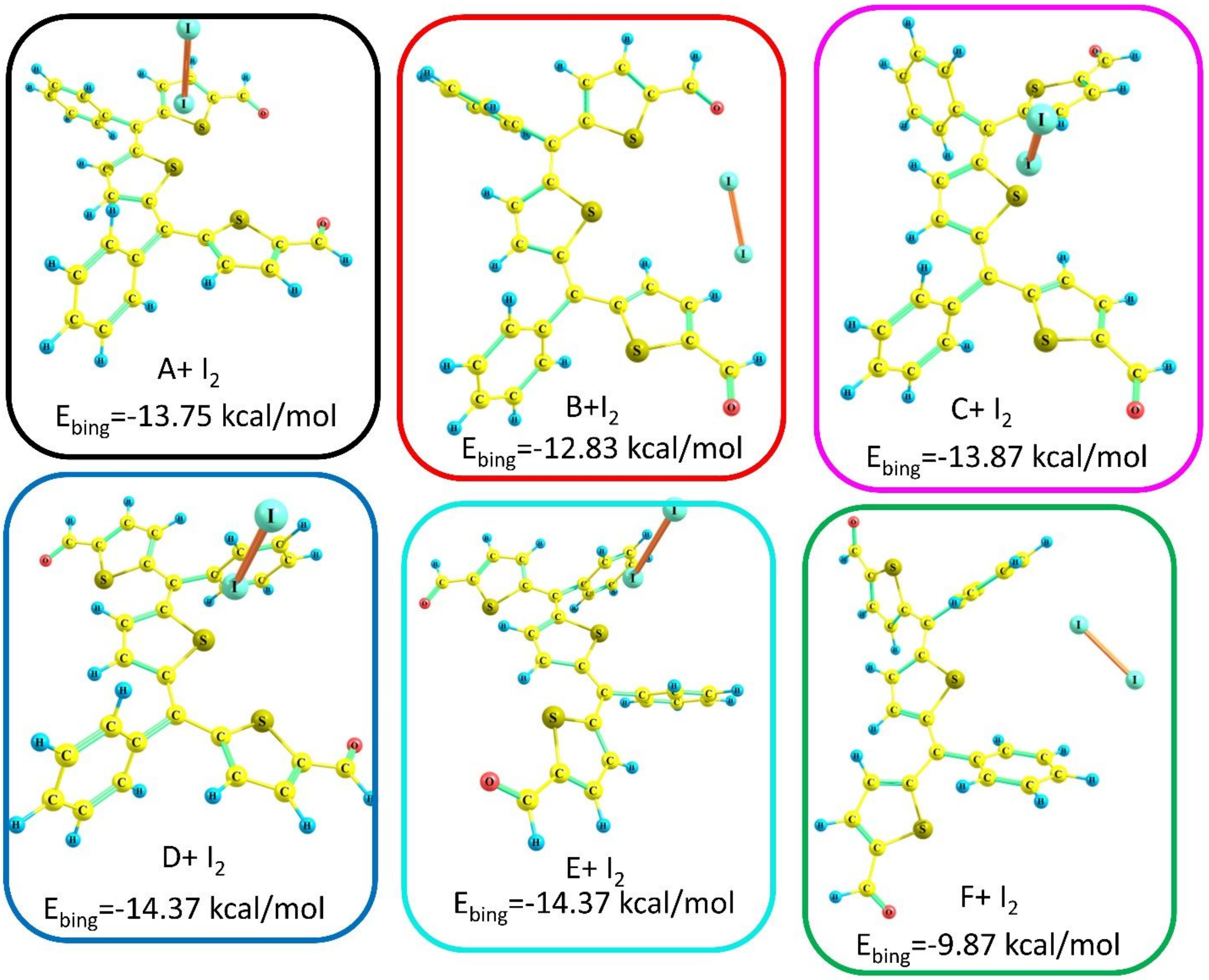

Figure S13. *Density functional theory calculations of the binding energies and optimized adducts of QOT building block isomers with* I_2*. The binding energy between isomer and iodine form was calculated using* $\Delta E = E_{tot} - E_{QOT} – E_{I2}$*, where* E_{QOT} *and* E_{I2} *are the energy of QOT and* I_2*, respectively, while* E_{tot} *is the energy of the adduct*

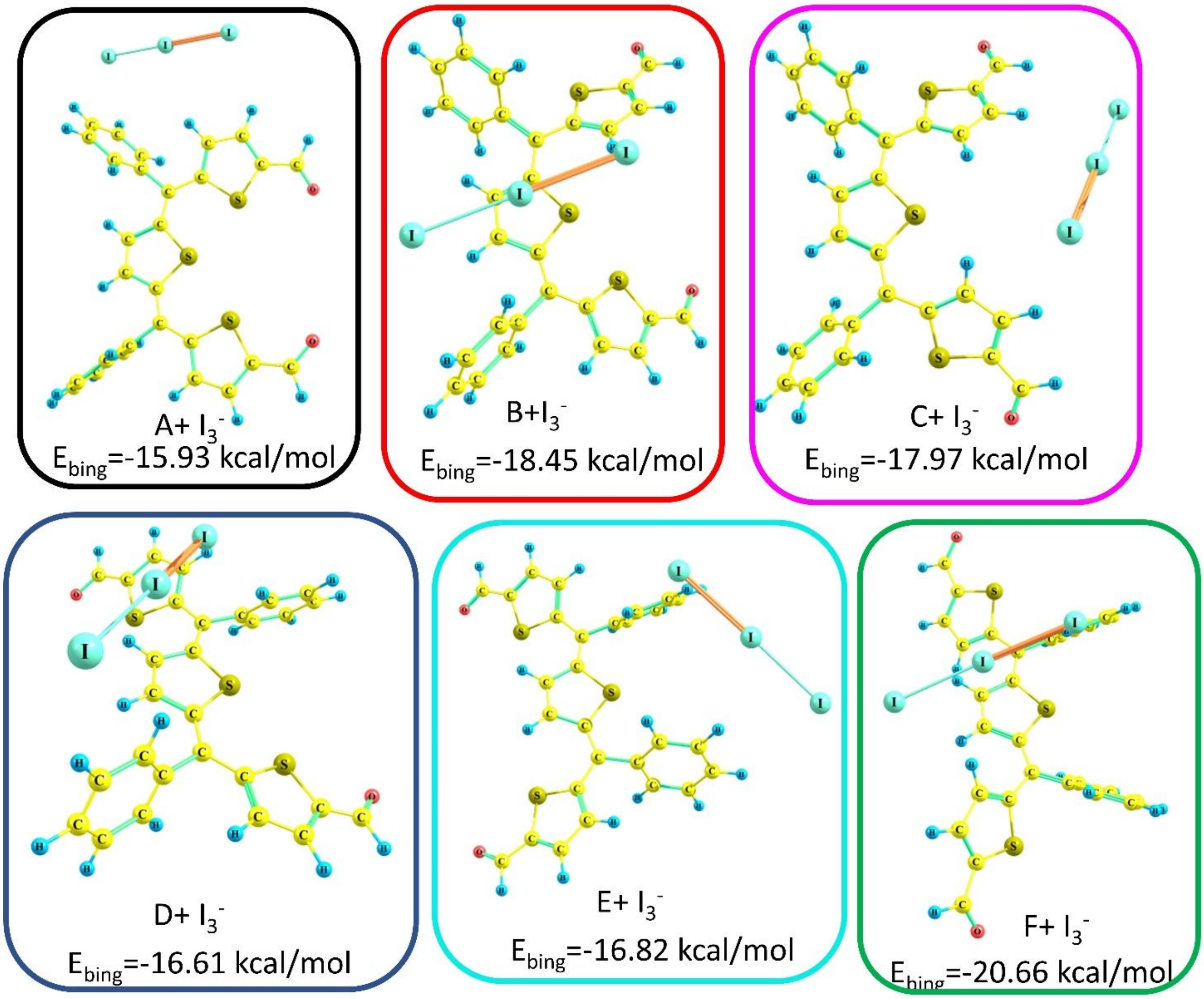

Figure S14. *Density functional theory calculations of the binding energies and optimized adducts of QOT building block isomers with* I_3^-.

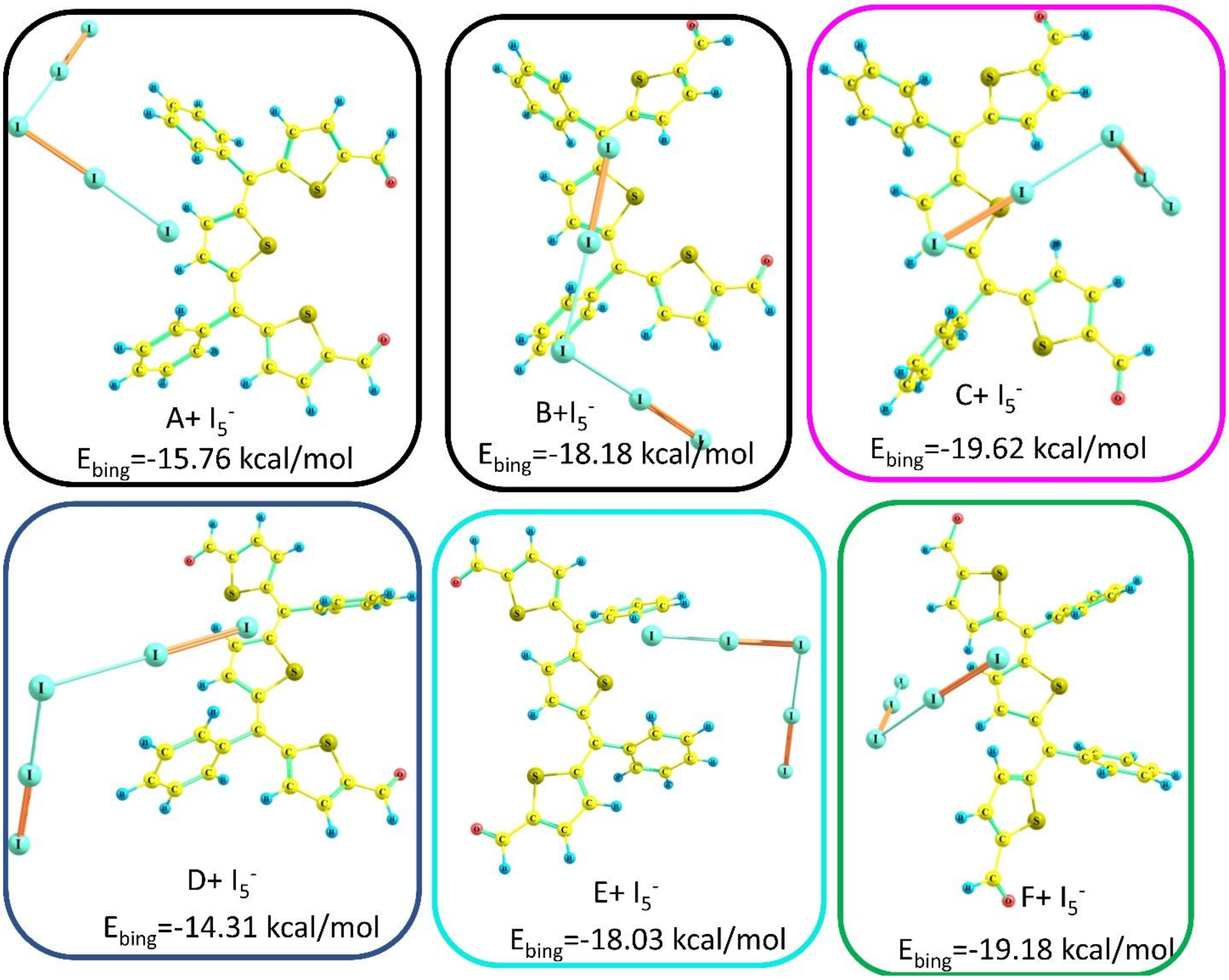

Figure S15. *Density functional theory calculations of the binding energies and optimized adducts of QOT building block isomers with* I_5^-.

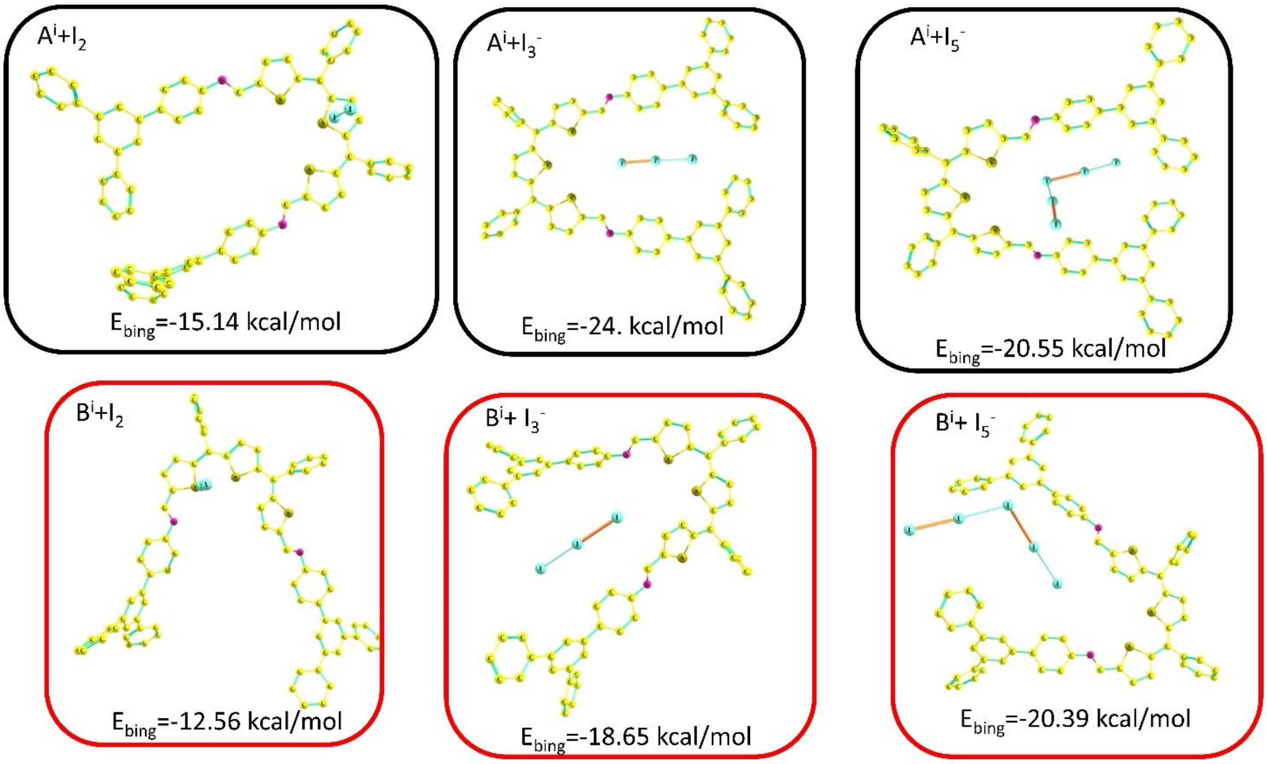

Figure S16*. Density functional theory calculations of the binding energies and optimized adducts of model compounds of QOT containing isomers (A^i and B^i) with I_2, I_3^- and I_5^-.*

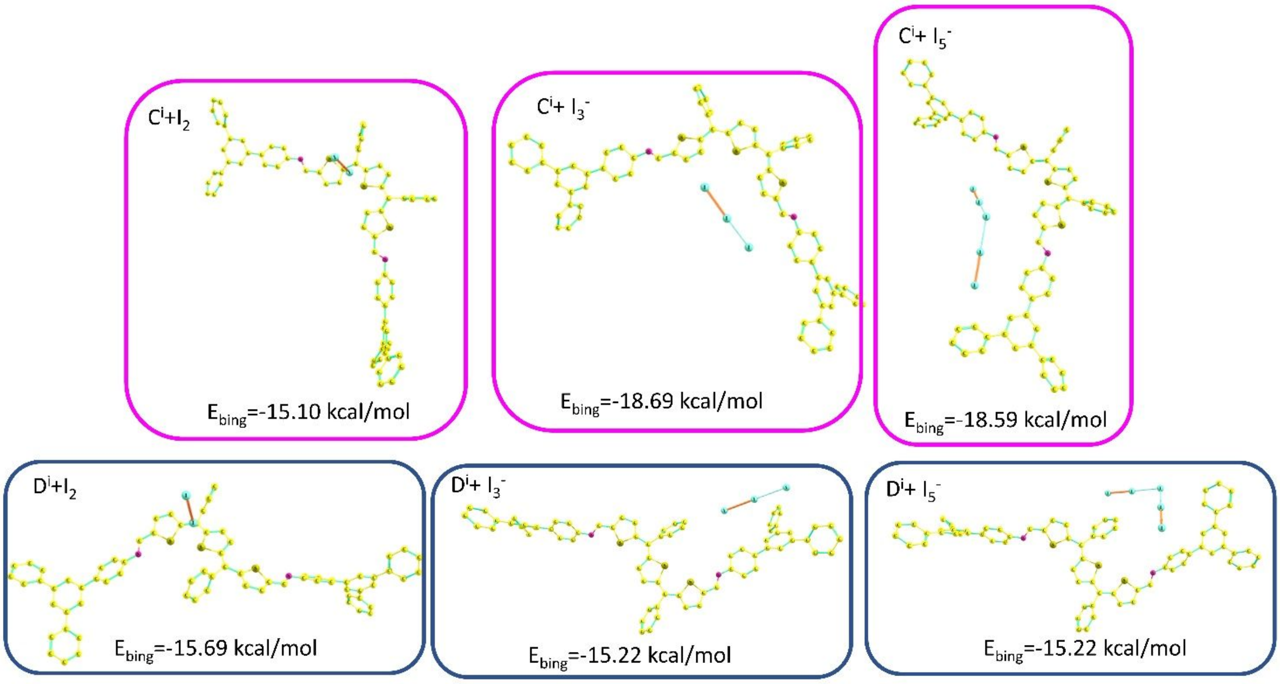

Figure S17. *Density functional theory calculations of the binding energies and optimized adducts of model compounds of QOT containing isomers (C^i and D^i) with I_2, I_3^- and I_5^-.*

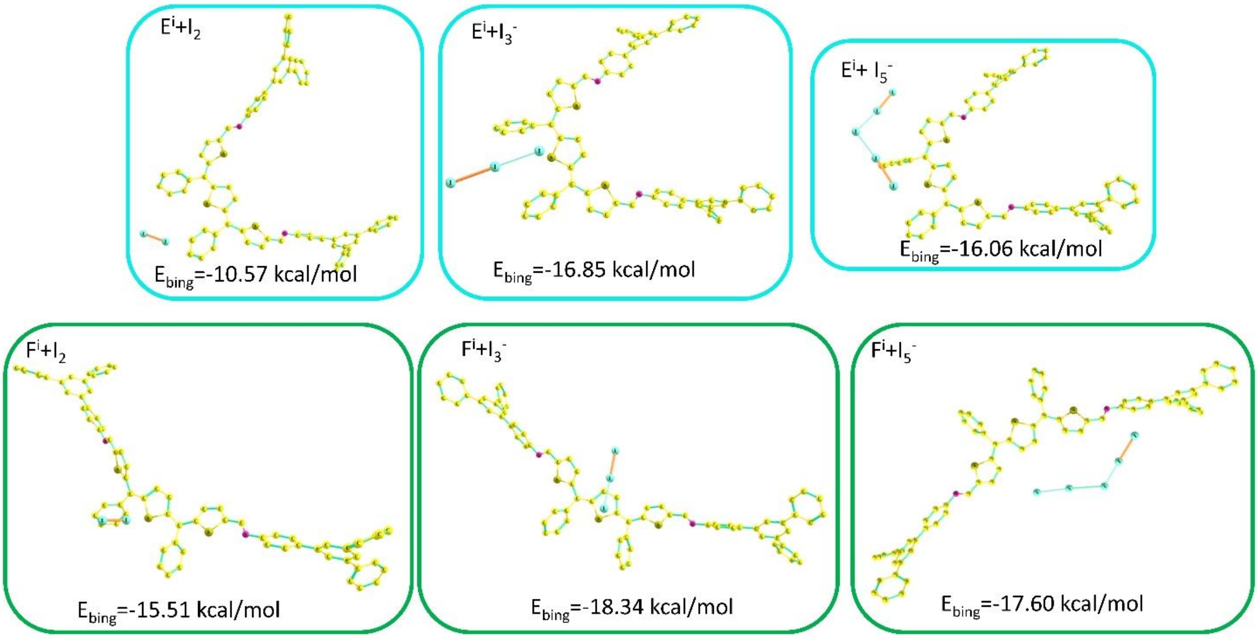

Figure S18. *Density functional theory calculations of the binding energies and optimized adducts of model compounds of QOT containing isomers (E^i and F^i) with I_2, I_3^- and I_5^-.*

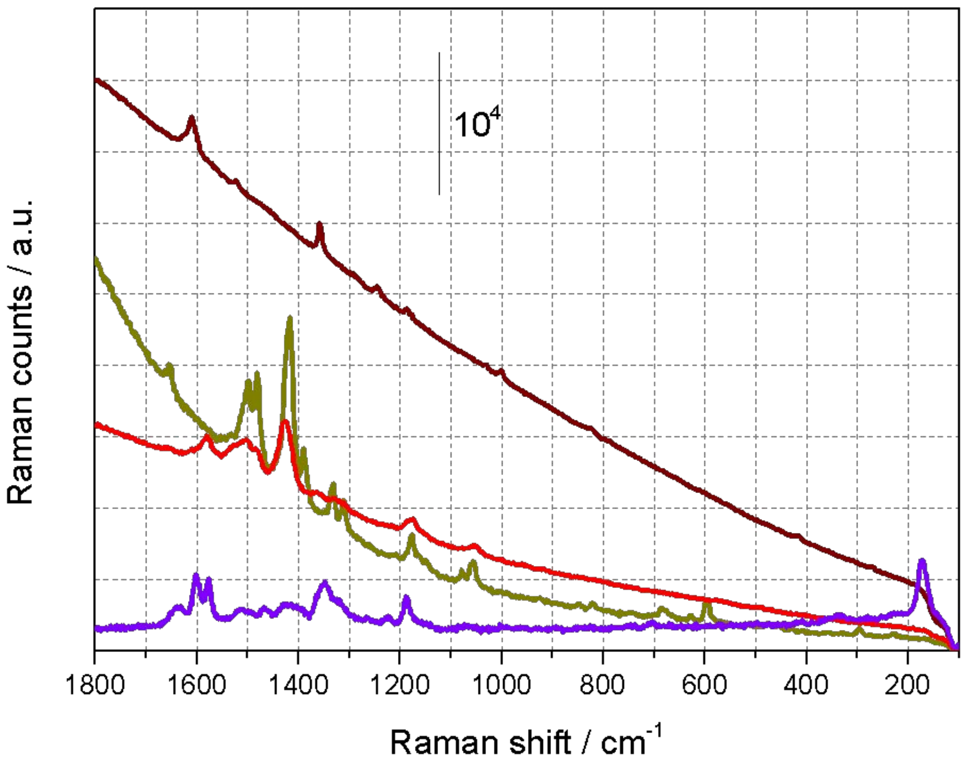

Figure S19: *Raman spectra recorded respectively on TABP (wine solid line), QOT (dark yellow solid line), pristine COF (red solid line) and COF-I_2 system (violet solid line, x4).*

Table S1. *Electronic charge distribution on selected atoms (sulfur and nitrogen) for pure and iodine-adsorbed isomers based on Mulliken population analysis.*

Isomer	S central thiophene ring	S left thiophene ring	S right thiophene ring	N from left side of the molecule	N from right side of the molecule	I from I_2 molecule
A’	-1.598	-1.198	-1.181	0.16	0.157	-
A’+I_2	-1.169	-1.259	-1.304	0.178	0.16	-0.188
B’	-1.7	-0.868	-1.071	0.124	0.133	-
B’+I_2	-1.331	-0.954	-0.777	0.151	0.127	-0.161
C’	-1.967	-0.851	-0.861	0.12	0.139	-
C’+I_2	-1.477	-0.793	-0.851	0.126	0.142	-0.196
D’	-1.537	-0.951	-1.012	0.119	0.13	-
D’+I_2	-0.948	-0.951	-1.024	0.134	0.133	-0.196
E’	-1.452	-1.013	-1.01	0.127	0.127	-
E’+I_2	-1.363	-0.967	-0.95	0.128	0.124	-0.102
F’	-1.295	-0.677	-0.677	0.13	0.13	-
F’+I_2	-0.804	-0.65	-0.696	0.132	0.132	-0.181

Table S2. *Electronic charge distribution on selected atoms (benzene carbons) for pure isomers based on Mulliken population analysis.*

Isomer	C1	C2	C3	C4	C5	C6
A’	Ring 1					
	1.114	-0.434	-0.074	-0.596	-0.257	-0.334
	Ring 2					
	1.218	-0.322	-0.348	-0.574	-0.185	-0.438
B’	Ring 1					
	1.111	-0.346	-0.273	-0.446	-0.268	-0.364
	Ring 2					
	1.114	-0.43	-0.198	-0.497	-0.217	-0.369

C’	Ring 1					
	1.114	-0.362	-0.24	-0.436	-0.25	-0.359
	Ring 2					
	1.108	-0.431	-0.203	-0.482	-0.224	-0.372
D’	Ring 1					
	1.092	-0.476	-0.189	-0.406	-0.271	-0.35
	Ring 2					
	1.142	-0.37	-0.299	-0.401	-0.218	-0.376
E’	Ring 1					
	1.139	-0.362	-0.289	-0.401	-0.229	-0.38
	Ring 2					
	1.202	-0.37	-0.206	-0.47	-0.265	-0.399
F’	Ring 1					
	1.25	-0.372	-0.224	-0.527	-0.207	-0.398
	Ring 2					
	1.118	-0.366	-0.222	-0.462	-0.257	-0.381

Table S3. *Electronic charge distribution on selected atoms (benzene carbons) for iodine-adsorbed isomers based on Mulliken population analysis.*

Adduct	C1	C2	C3	C4	C5	C6
A’+I_2	Ring 1					
	1.135	-0.435	-0.107	-0.575	-0.258	-0.34
	Ring 2					
	1.152	-0.264	-0.301	-0.602	-0.308	-0.373
B’+I_2	Ring 1					
	1.117	-0.353	-0.226	-0.421	-0.403	-0.361
	Ring 2					
	1.113	-0.423	-0.201	-0.508	-0.192	-0.376
C’+I_2	Ring 1					
	1.108	-0.371	-0.221	-0.504	-0.205	-0.427

	**Ring 2**					
	1.149	-0.361	-0.254	-0.45	-0.232	-0.362
**D'+I_2**	**Ring 1**					
	1.085	-0.46	-0.195	-0.347	-0.285	-0.385
	**Ring 2**					
	1.135	-0.376	-0.301	-0.424	-0.208	-0.368
**E'+I_2**	**Ring 1**					
	1.144	-0.366	-0.291	-0.399	-0.228	-0.38
	**Ring 2**					
	1.213	-0.372	-0.218	-0.456	-0.256	-0.402
**F'+I_2**	**Ring 1**					
	1.249	-0.377	-0.22	-0.521	-0.201	-0.4
	**Ring 2**					
	1.189	-0.384	-0.249	-0.47	-0.218	-0.377

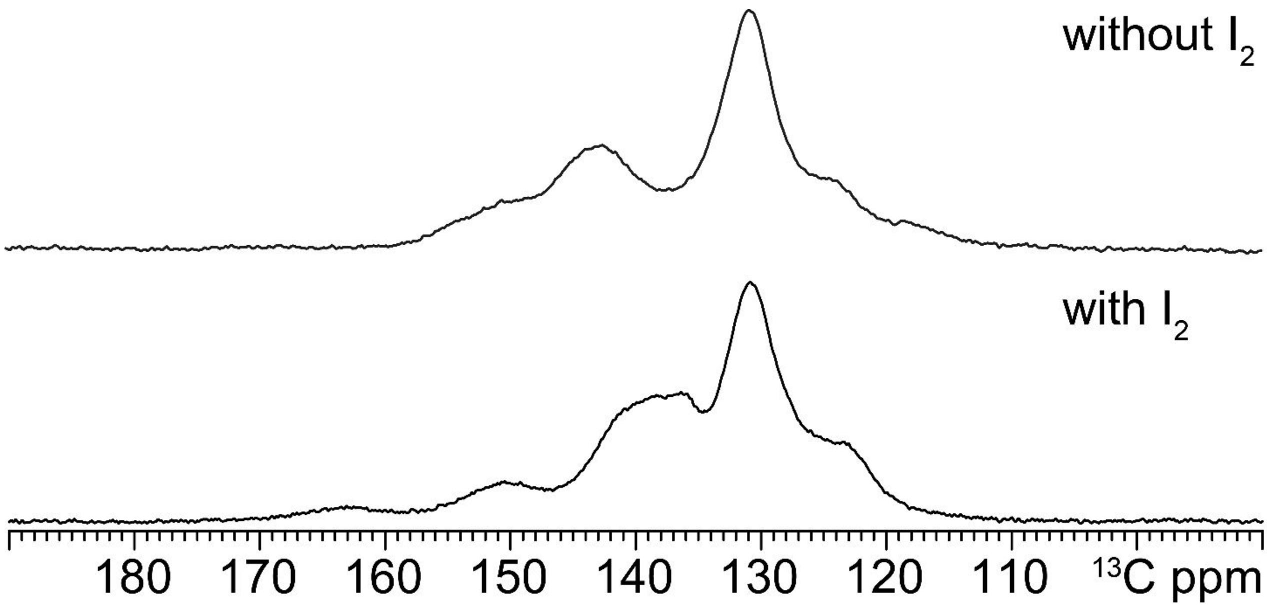

Figure S20. *Stacked plot of ^{13}C (150.91 MHz) CPMAS (20 kHz) NMR spectra of TAPB-QOT-COP free (bottom) and TABP-QOT-COP loaded with I_2 (top).*

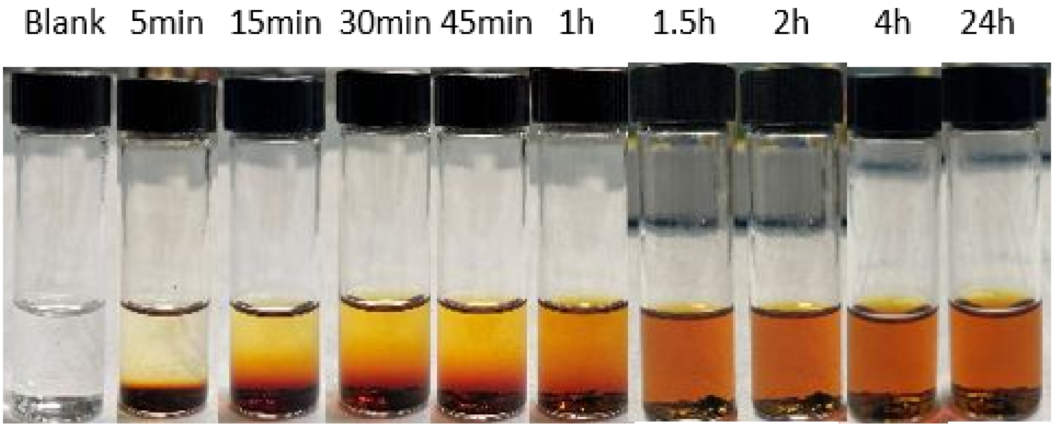

Photo S2 Photographs indicating the iodine release of iodine-adsorbed TPB-QOT COP in ethanol.

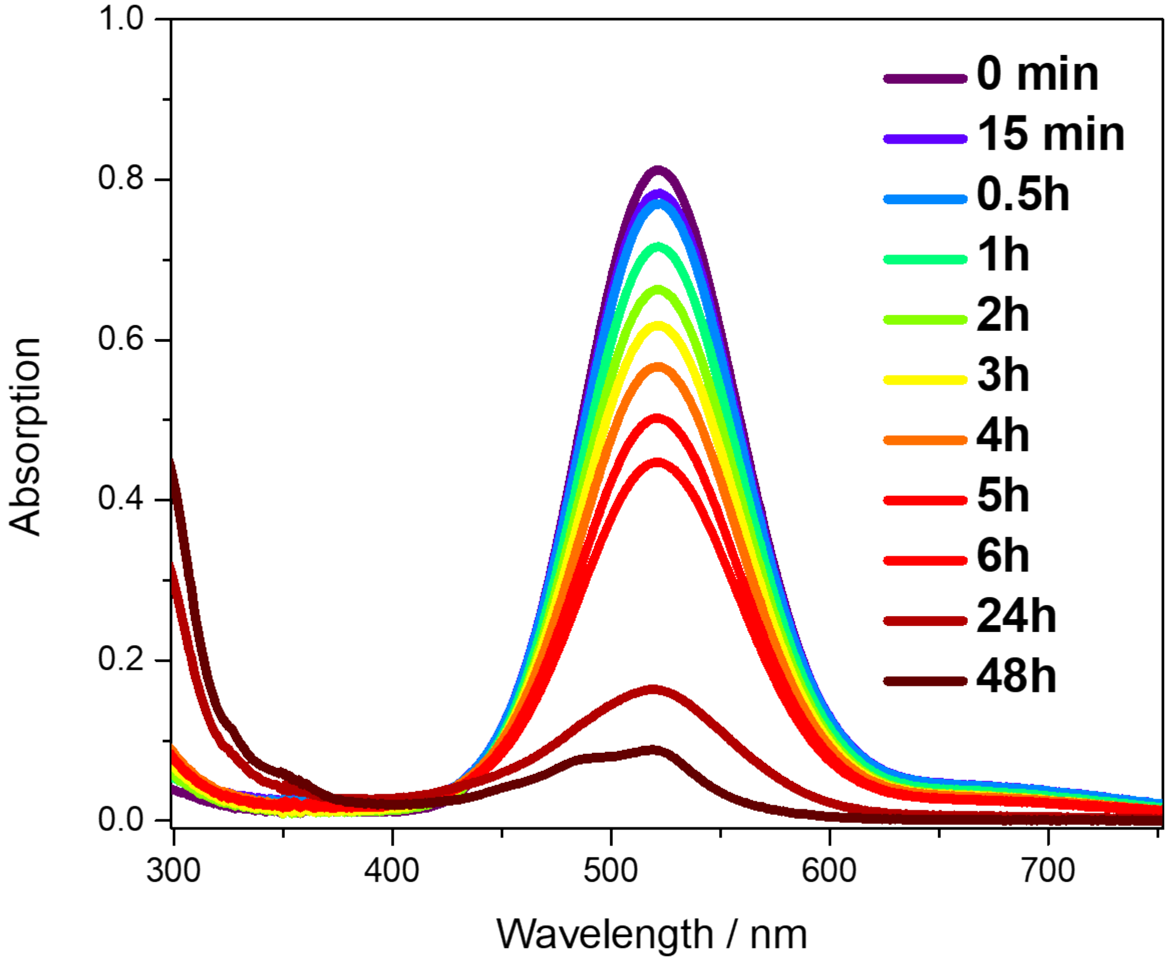

Figure S21. *UV−vis spectra for TPB-QOT COP (5 mg) and Iodine (20 mg/2ml) in n-hexane solution.*

APPENDIX A

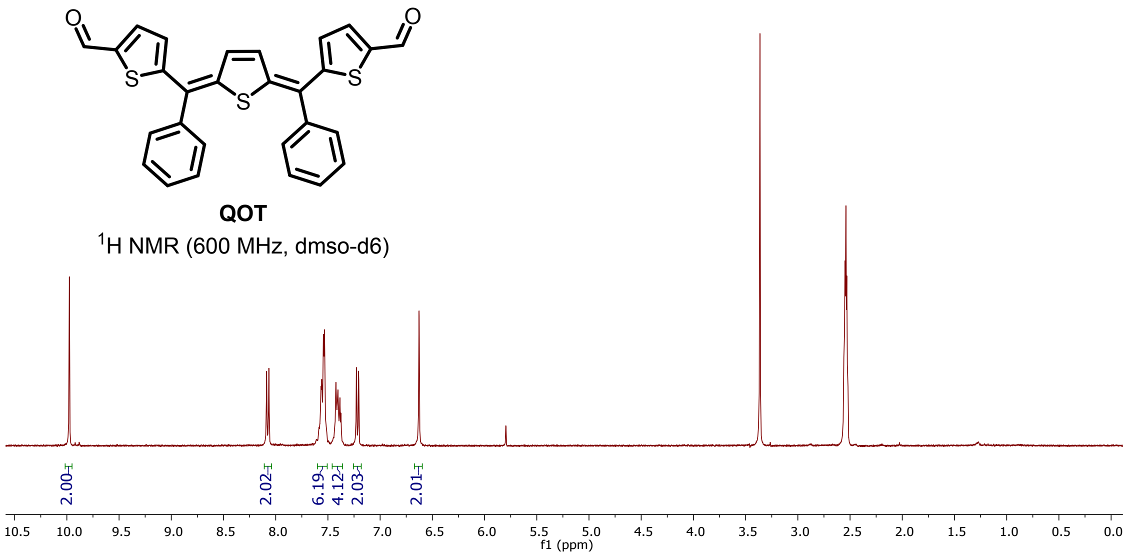

QOT
^{1}H NMR (600 MHz, dmso-d6)
2.00
2.02
6.19
4.12
2.03
2.01
f1 (ppm)

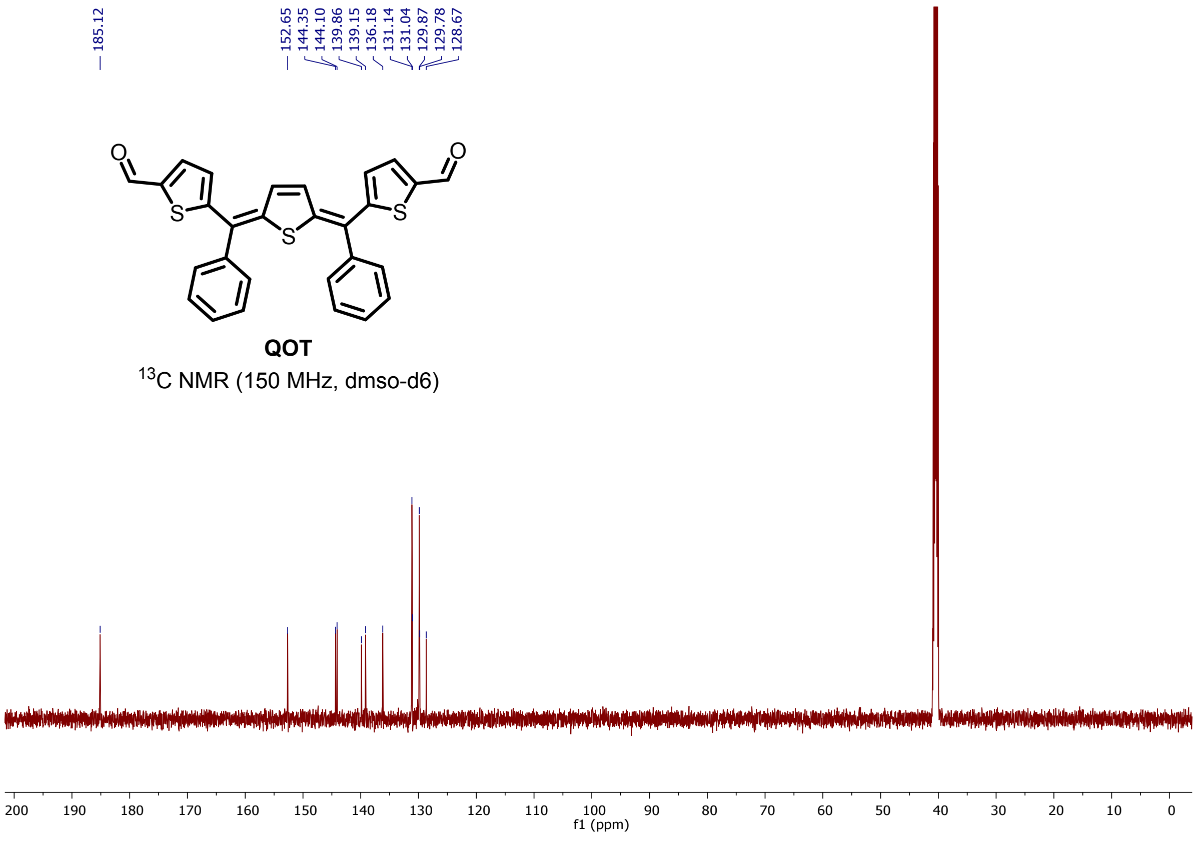

185.12
152.65
144.35
144.10
139.86
139.15
136.18
131.14
131.04
129.87
129.78
128.67
QOT
^{13}C NMR (150 MHz, dmso-d6)
f1 (ppm)

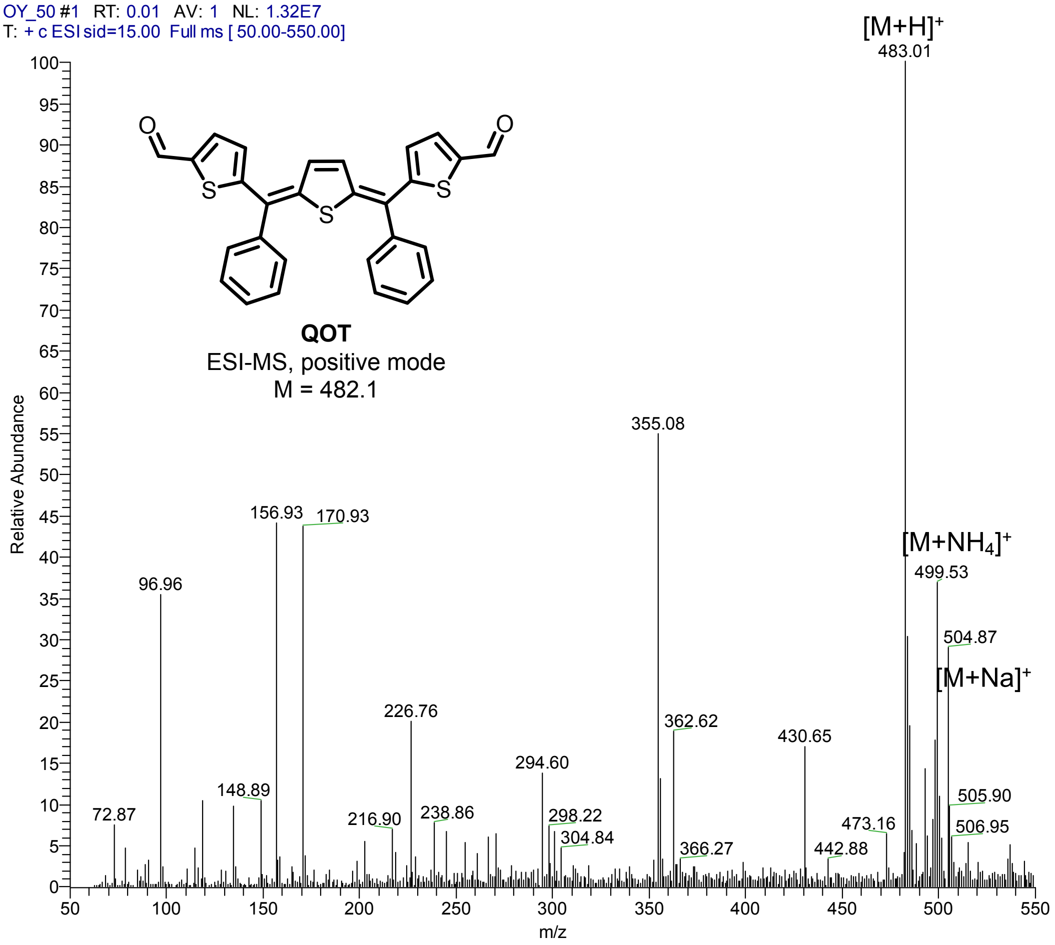
OY_50 #1 RT: 0.01 AV: 1 NL: 1.32E7
T: + c ESI sid=15.00 Full ms [50.00-550.00]
QOT
ESI-MS, positive mode
M = 482.1
[M+H]+
483.01
[M+NH4]+
499.53
504.87
[M+Na]+
355.08
156.93
170.93
96.96
226.76
362.62
430.65
294.60
148.89
72.87
216.90
238.86
298.22
304.84
366.27
442.88
473.16
505.90
506.95
Relative Abundance
m/z

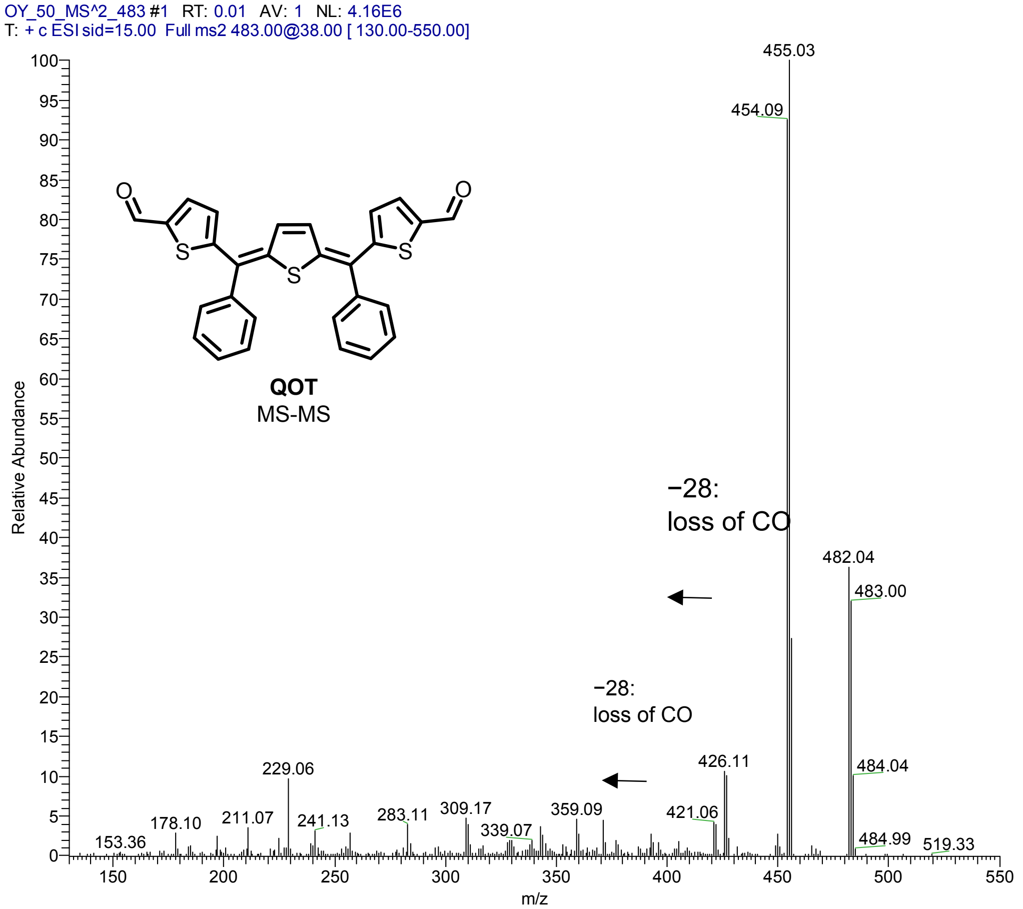
OY_50_MS^2_483 #1 RT: 0.01 AV: 1 NL: 4.16E6
T: + c ESI sid=15.00 Full ms2 483.00@38.00 [130.00-550.00]
QOT
MS-MS
455.03
454.09
−28:
loss of CO
482.04
483.00
−28:
loss of CO
426.11
484.04
229.06
153.36
178.10
211.07
241.13
283.11
309.17
339.07
359.09
421.06
484.99
519.33
Relative Abundance
m/z

APPENDIX B

Materials

Thiophene (TCI, >98.0%, CAS No: 110-02-1), benzoic acid (Sigma Aldrich, ≥99.5%, CAS No: 65-85-0), trifluoroacetic anhydride (TFAA) (Sigma Aldrich, 99%, CAS No: 407-25-0), trifluoroacetic acid (TFA) (Sigma Aldrich, ≥99.0%, CAS No: 76-05-1), acetic acid(Sigma Aldrich, ≥99.7%, CAS No: 64-19-7), *N,N,N',N'*-tetramethylethylenediamine (TMEDA) (Sigma Aldrich, 99%, CAS No.: 110-18-9), *n*-butyllithium (1.6 M solution in hexane) (Sigma Aldrich, CAS No.: 109-72-8), sodium hydrosulphite ($Na_2S_2O_4$) (Sigma Aldrich, ≥82%, CAS No.: 7775-14-6), hydriodic acid (57% w/w in distilled water) (Sigma Aldrich, CAS No.: 10034-85-2), phosphorus(V) oxychloride ($POCl_3$) (Sigma Aldrich, 99%, CAS No.: 10025-87-3), anhydrous *N,N*-dimethylformamide (DMF) (Sigma Aldrich, 99.8%, CAS No.: 68-12-2), sodium hydroxide (NaOH) (Sigma Aldrich, ≥98%, pellets, CAS No.: 1310-73-2), anhydrous sodium sulphate (Na_2SO_4) (Sigma Aldrich, ≥99.0%, powder, CAS No.: 7757-82-6) and solvents were used without further purification. The reaction of linkers and building blocks were monitored by thin-layer chromatography (TLC) performed on silica gel TLC-PET foils GF 254, particle size 25 mm, medium pore diameter 60 Å.

Methods

The solution-state ^1H and ^{13}C NMR spectra were recorded on a JEOL ECZR 600 (^1H NMR operating frequency 600 MHz) at 298 K, in DMSO-d6, using the residual solvent peak as internal reference (^1H: $(CH_3)_2SO$ 2.54 ppm; ^{13}C: $(CD_3)_2SO$ 40.45 ppm). Mass spectra were recorded using an LCQ Advantage MAX Ion Trap Spectrometer (Thermo Fisher Scientific, Dreieich, Germany) equipped with an electrospray ion source. Flash column chromatography was performed on Biotage Isolera™ system. UV-Vis absorption spectra were measured with a double-beam Perkin–Elmer Lambda 20 UV-Vis spectrophotometer equipped with a 1-cm quartz cell. Solid state UV-Vis measurements were carried on Cary 5000 UV-Vis-NIR spectrometer from Agilent. Vibrational IR spectra were collected in ATR mode on the sample powder using a Thermo Scientific Nicolet 6700 spectrometer. Elemental analysis was performed using a Thermo Nicolet FlashEA 1112 Series.

Powder X-ray diffraction (PXRD) patterns were taken with a Panalytical X'Pert PRO MPD diffractometer equipped with a $Cu(K_\alpha)$ source operating in reflectance Bragg-Brentano geometry employing Ni filtered $Cu(K_\alpha)$ line focused radiation at 1600 W (45 kV, 40 mA) power.

The solid-state NMR spectra were acquired with a JEOL ECZR 600 instrument, operating at 600.17, 150.91 and 60.82 MHz for ^1H, ^{13}C and ^{15}N nuclei, respectively. The sample was packed into cylindrical zirconia rotors with a 3.2 mm o.d. and a 60 mL volume. All the powder was used, without further preparations, to fill the rotor. All experiments were acquired at 20 kHz spinning speed at room temperature except for the ^{15}N CPMAS spectrum that was acquired at a spinning speed of 12 kHz.

^{13}C and ^{15}N CPMAS spectra were acquired using a ramp cross-polarization pulse sequence with a 90° ^{1}H pulse of 2 μs, contact times of 3.5 (^{13}C) and 7 ms (^{15}N), and an optimized recycle delay of 2 s for TAPB-QOT-COP, 13 s for QOT, and 60 s for TAPB. For the ^{13}C CPMAS spectrum, a two-pulse phase modulation decoupling scheme was used, with a radiofrequency field of 69.4 kHz. Instead for ^{15}N CPMAS spectrum, a SPINAL64 decoupling was used, with a radiofrequency field of 45.6 kHz. For ^{13}C CPMAS spectra we acquired 1320 scans for TAPB-QOT-COP, 160 scans for QOT and 64 scans for TAPB (time domain in the direct dimension: 2048). The ^{15}N CPMAS spectrum was acquired with 475 scans (time domain in the direct dimension: 1400) for the TAPB molecule. ^{13}C and ^{15}N chemical shift scales were referenced with the resonance of α-glycine (^{13}C methylene signal at 43.5 ppm), and $(NH_4)_2SO_4$ (^{15}N signal at 24.6 ppm with respect to NH_3), respectively, as external standards.

A Micromeritics ASAP 2020 volumetric apparatus was used to measure both N_2 and CO_2 adsorption isotherms at 77 K and 273 K, respectively from vacuum up to 1100 mbar. Prior to the measurement, powders were degassed overnight at 373 K reaching a residual pressure of 10^{-4} mbar. Measurements with CO_2 at 273 K were collected employing a home-made patented apparatus consisting in a custom quartz cell equipped with a plug-in thermal jacket for measurements in thermostatic fluid,[1] coupled with the Micromeritics ASAP 2020 analyzer. The sample temperature of the cell was kept constant during the adsorption measurement using an external isothermal liquid bath

(Julabo F25-EH). Specific surface areas (SSAs) were determined by applying the Brunauer–Emmett–Teller (BET) model to the CO_2 adsorption isotherm in the $0.02<p/p^0<0.03$ range. The cumulative pore volume and pore size distribution were obtained by applying the Non Localized Density Functional Theory (NL-DFT) method to the CO_2 adsorption isotherm at 273 K, considering a slit pore geometry and applying a model for CO_2 adsorption at 273 K on carbons provided by Micromeritics and a medium-low regularization.

SEM measurements were performed using FESEM TESCAN S9000G microscope (Microanalysis; OXFORD - Detector Ultim Max - Software AZTECT) equipped with Schottky emitter source (Resolution: 0.7 nm at 15keV in beam mode, using accelerating voltages in the range of 0.2-30 keV). Before measuring the samples have been sputtered with 20 nm of gold.

Thermogravimetric analysis (TGA) data was recorded with a TA instruments Q600 thermobalance in dry N_2 flow (100 mL/min) with a ramp of 10 K/min from 300 to 1073 K.

Raman spectra have been collected on an inVia Raman Microscope and adopting a 514 nm Ar^+ exciting LASER line. LASER light/backscattered light have been focused/collected on/from samples through an Olympus 20x ULWD objective (NA = 0.40). In the case of solid powders, 0.5% of the total LASER power has been admitted reaching the samples. For solutions (measured in a Helma QS cuvette and subjected to a magnetic stirring), 10% of the total laser power has been adopted. In any case, stability of samples under the LASER light have been carefully investigated. Backscattered light

(after Rayleigh light removal through an edge filter) have been analysed by an 1800 l/mm grating and collected through a CCD Peltier cooled detector. For powders, each presented spectrum is resulting from the average of spectra (30 x 20" acquisitions) collected on three different points. For solutions, the presented spectra results from the average of three spectra recorded consecutively (20 x 20" acquisitions).

The iodine uptake/release kinetic was investigated as follows: TAPB-QOT polymer (10 mg) was placed into a small open vial and the whole system was weighed; then, this small vial was placed into a larger one where excess iodine solid (500 mg) were added, and the whole system was closed (Photo S2). Then, the system was heated in an oven at 348 K to promote the I_2 sublimation. After a fixed amount of time, it was removed, cooled to room temperature, and the small vial was weighed, comparing its weight before and after iodine adsorption. The following equation calculated the I_2-loading weight of samples: $\alpha = (m_2-m_1)/m_1*100$, being α the iodine uptake, m_1 the mass of polymer before adsorption of Iodine and m_2 = the mass of polymer after adsorption of Iodine). To confirm the reproducibility of the approach, adsorption studies were repeated 5 times (for five nominally identical aliquots) in the same experimental conditions.

Prior to using the TAPB-QOT polymer in recycling studies, iodine was released by immersing the material (5 mg) in EtOH (3 ml) and stirring vigorously on a plate at room temperature. This process continued until the solvent was transparent (240 minutes were enough to assure maximum I_2 release).

Then, the polymer was re-activated under vacuum oven at 393 K to remove solvent entrapped within the pores. This process was repeated 5 times. Iodine adsorption was also performed in a hexane solution (*i.e.* liquid phase): 5 mg of the polymer was added to an iodine solution of hexane (20 mg/2 ml). Uptake kinetics were monitored by UV-Vis absorption and for each measurement the adsorption capacity was determined according to the Beer-Lambert Law equation.

Computational methods

All density functional calculations presented here were performed with the Gaussian 09 program.[2] The ground-state geometries were fully optimized using a DFT level without any symmetry constrains using B3LYP hybrid exchange-correlation functional[3] and 6-311++G(d,p) basis set for QOT and TAPB-QOT systems and 6-31G basis set for COF structural unit. All found stationary points were identified as minima (no normal vibrations with imaginary frequency were detected). The graphical representations of the molecular structures were made using ChemCraft software (Version 1.6).[4]

The binding energy was calculated as follows:

$$E_{binding} = (E_{unit + I_n} - E_{unit} - E_{I_n}) \quad (1)$$

Where E_{unit} is the total energy of the studies isomer, E_{In} is the total energy of iodine form, $E_{unit+In}$ is the total energy of the corresponding iodine absorbing isomer system. At the beginning of modeling,

the geometry of the isomer unit+iodine form was optimized by using B3LYP/6-311++G(d,p) and LanL2DZ for the iodine atom level of theory. Further, the one-shot calculation was done with the dispersion corrected density functional theory (D3BJ) correction and obtained the total energy used for binding energy calculation.[5] All total energy in equation 1 is used with D3BJ correction.

Synthetic procedures

Synthesis of 5,5'-((1*E*,1'*E*)-thiophene-2,5-diylidenebis(phenylmethaneylylidene))bis(thiophene-2-carbaldehyde), (QOT) – Scheme S1.

Synthesis of phenyl(thiophen-2-yl)methanone (1):[6]

A 100 ml one neck flask was charged with benzoic acid (4.14 g, 34 mmol), thiophene (4.29 g, 4.08 mL, 50.96 mmol) TFAA (16 ml) and TFA (10.8 ml). The reaction mixture was stirred at room temperature for 24 h and monitored by TLC. Solution was straw-coloured and after 1 hour the colour turned dark blue-green. Upon completion, the excess of TFAA and TFA was distilled. The mixture was extracted with DCM, dried over Na_2SO_4, filtered, the solvent was removed and the residue was recrystallized from petroleum ether (4.6 g, 72%). ^{1}H NMR (600 MHz, DMSO-d6) δ (ppm): 8.08 – 8.03 (m, 1H), 7.80 – 7.72 (m, 2H), 7.68 – 7.63 (m, 1H), 7.63 – 7.56 (m, 1H), 7.55 – 7.45 (m, 2H), 7.25 – 7.19 (m, 1H). GC-MS: m/z = 188 $[M + H]^+$.

Synthesis of (2*E*,5*E*)-2,5-bis(phenyl(thiophen-2-yl)methylene)-2,5-dihydrothiophene (3):[7]

To a 50 mL two-neck flask, anhydrous hexane (25 mL), *N,N,N',N'*-tetramethylethylenediamine (TMEDA) (1.55 g, 2.0 mL, 13.3 mmol), thiophene (0.45 g, 0.43 mL, 5.4 mmol), *n*-butyllithium (8 ml, 12.8 mmol) were added dropwise to the stirred solution at room temperature. The reaction mixture was refluxed for 1 h and then cooled to −40 °C. Then, a solution of phenyl thienyl ketone **1** (2.44 g, 13 mmol) in anhydrous diethyl ether (25 ml) was added dropwise to the reaction mixture. The mixture was warmed up to room temperature and stirred overnight. The reaction was then quenched with 1 M NH_4Cl_{aq} (20 mL) and the organic phase was extracted with chloroform and ethyl acetate. Finally, the combined organic phases were dried over Na_2SO_4, filtered, and the solvent was evaporated under vacuum to afford thiophene-2,5-diylbis(phenyl(thiophen-2-yl)methanol) (**2**), which was used for the next step without further purification. The obtained crude alcohol product (**2**) was dissolved in toluene and a solution of $Na_2S_2O_4$ (13.8 g, 79 mmol) and 57% HI (13.5 mL) in distilled water (50 mL) was added. The two-phase system was vigorously stirred at room temperature for 24 h. An orange-coloured reaction mixture was neutralized with $NaHCO_3$ and extracted several times with diethyl ether. The combined organic phases were dried over Na_2SO_4 and filtered. After evaporation of the solvent, the product was purified through flash column chromatography on silica column (DCM/PE 1/5), obtaining pure product **3** (1.24 g, 45 %) ^{1}H NMR (600 MHz, DMSO-d6) δ (ppm): 7.63 (dd, J = 5.0, 1.1 Hz, 2H), 7.48 – 7.32 (m, 6H), 7.32 – 7.16 (m, 4H), 7.09 (dd, J = 5.0, 3.8 Hz, 2H), 6.94 (dd, J

= 3.8, 1.1 Hz, 2H), 6.36 (s, 2H), UV-Vis: λ_{max} (THF) = 470 nm, FT-IR (cm^{-1}): 3098, 3059, 3019, 1599, 1540, 1481, 1488, 1436, 1141, 1076, 1023, 807, 749, 690

Synthesis of 5,5'-((1*E*,1'*E*)-thiophene-2,5-diylidenebis(phenylmethaneylylidene)) bis(thiophene-2-carbaldehyde) (QOT)

$POCl_3$ (1.64 g, 10.73 mmol, 1 mL) was added dropwise into a solution of compound 3 (1.45 g 3.38 mmol) in DMF (1.2 ml) in a flame-dried reaction flask, kept below 10 °C. The mixture was then stirred for 30 min at room temperature before being heated at 90–95 °C for 90 min. After cooling down, the mixture was poured into crushed ice (50 mL) and made weakly alkaline with a NaOH solution (1 M). After partitioning between DCM and water, the organic phases were dried over Na_2SO_4 and filtered. The solvent was removed under vacuum, dried at room temperature, affording **QOT** as the product as a mixture of isomers (1 g, 95%) that were transformed into a single one in a vacuum oven at 373 K for one night. ^1H NMR (600 MHz, DMSO-d6) δ (ppm): 9.86 (s, 2H), 7.96 (d, J = 4.3 Hz, 2H), 7.48 – 7.37 (m, 6H), 7.28 (dd, J = 6.5, 2.8 Hz, 4H), 7.10 (d, J = 4.1 Hz, 2H), 6.51 (s, 2H), ^{13}C NMR (150 MHz, DMSO-d6) δ (ppm): 185.1, 152.7, 144.4, 144.1, 139.9, 139.2, 136.2, 131.1, 131.0, 129.9, 129.8, 128.7, LC-ESI-MS (*m/z*): 483.01 $[M+H]^+$, 499.53 $[M+NH_4]^+$, 504.87 $[M+Na]^+$; UV-Vis: λ_{max} (THF) = 523 nm; FT-IR (cm-1): 3070, 2824, 1650, 1499, 1406, 1220, 1132, 1038, 796, 750, 698, 664, 617.; Elemental Analyses for $C_{28}H_{18}O_2S_3$. Calculated: C, 69.68; H, 3.76; S, 19.93; C:S ratio, 3.50. Found: C, 67.98, H, 3.23; S, 19.52; C:S ratio, 3.48.

Synthesis of quinoid oligothiophene-containing 2D covalent organic polymer (TAPB-QOT-COP) -Figure 1a (Main text)

To a pre-dried vial, TAPB and QOT were added and the vial was sealed off. The air was evacuated by argon using a needle from the sealed vial. Solvent mixtures (dioxane/mesitylene, 4 mL, 1/1 in vol.) were added via syringe (solvents were degassed by three freeze-pump-thaw cycles before using). Suspended solution was degassed by three freeze-pump-thaw cycles for 3 times. Monomers were dissolved via sonication and then 6M CH_3COOH (0.1 ml) was added. The vial was sealed off and heated in an oil bath or in an oven at a certain time for 5 days without stirring. The precipitate was filtered by vacuum, washed with anhydrous dioxane, THF, and diethyl ether several times, and dried under vacuum oven at 393 K for 24 h, obtaining a dark-violet powder (60%). Elemental Analyses for $C_{44}H_{28}N_2S_3$: C, 77.61; N, 4.11; H, 4.14; S, 14.14, C:N; 18.8. Found: C, 72.69; N, 3.93; H, 4.02; S, 11.90, C:N; 18.5.

References

1 PCT/IB2021/051769, WO2021/181211A12021, 2021.

2 Frisch, M. J.; Trucks, G. W.; Schlegel, H. B.; Scuseria, G. E.; Robb, M. A.; Cheeseman, J. R.; Scalmani, G.; Barone, V.; Mennucci, B.; Petersson, G. A.; Nakatsuji, H.; Caricato, M.; Li, X.; Hratchian, H. P.; Izmaylov, A. F.; Bloino, J.; Zheng, G.; Sonnenberg, J. L.; Hada, M.; Ehara, M.; Toyota, R.; Fukuda, K.; Hasegawa, J.; Ishida, M.; Nakajima, T.; Honda, Y.; Kitao, O.; Nakai, H.; Vreven, T.; Montgomery, J. A. Jr.; Peralta, J. E.; Ogliaro, F.; Bearpark, M.; Heyd, J. J.; Brothers, E.; Kudin, K. N.; Staroverov, V. N.; Kobayashi, R.; Normand, J.; Raghavachari, K.; Rendell, A.; Burant, J. C.; Iyengar, S. S.; Tomasi, J.; Cossi, M.; Rega, N.; Millam, M. J.; Klene, M.; Knox, J. E.; Cross, J. B.; Bakken, V.; Adamo, C.; Jaramillo, J.; Gomperts, R.; Stratmann, R. E.; Yazyev, O.; Austin, A. J.; Cammi, R.; Pomelli, C.; Ochterski, J. W.; Martin, R. L.; Morokuma, K.; Zakrzewski, V. G.; Voth, G. A.; Salvador, P.; Dannenberg, J. J.; Dapprich, S.; Daniels, A. D.; Farkas, Ö.; Foresman, J. B.; Ortiz, J. V.; Cioslowski, J.; Fox, D. J.; Gaussian, I.; Caricato, M.; Marenich, A.; Bloino, J.; Janesko, B. G.; Gomperts, R.; Mennucci, B.; Hratchian, H. P.; Ort, J. V.; Fox, D. J. *Gaussian*

09 (Gaussian, Inc., Wallingford CT, 2009).

3 Lee, C.; Yang, W.; Parr, R. G. Development of the Colle-Salvetti correlation-energy formula into a functional of the electron density, *Phys. Rev. B* **1988**, *37*, 785–789.

4 Zhurko, G. A.; http://www.chemcraftprog.com. Accessed on January 2023.

5 Grimme, S.; Ehrlich, S.; Goerigk, L. Effect of the damping function in dispersion corrected density functional theory, *J. Comput. Chem.* **2011**, *32*, 1456–1465.

6 Liu, G.; Xu, B. Hydrogen bond donor solvents enabled metal and halogen-free Friedel–Crafts acylations with virtually no waste stream, *Tetrahedron Lett.* **2018**, *59*, 869–872.

7 Umeyama, T.; Watanabe, Y.; Oodoi, M.; Evgenia, D.; Shishido, T.; Imahori, H. Synthesis of low bandgap polymers based on thienoquinodimethane units and their applications in bulk heterojunction solar cells, *J. Mater. Chem.* **2012**, *22*, 24394–24402.
